# Glaucoma Filtration Surgery in Rabbit Pre-Clinical Models: Design, Drug Dosing and Reporting Practices 2011–2025

**DOI:** 10.3390/vision10030056

**Published:** 2026-08-19

**Authors:** Maryam Khan, Liam Bourke, Adrián M. Alambiaga-Caravaca, Tauseef Ahmad, Mark Lemoine, Iluore Asekomhe, Golestan Salimbeigi, Nina Pohler, Colm O’Brien, Alan J. Hibbitts

**Affiliations:** 1 Tissue Engineering Research Group, Department Anatomy & Regenerative Medicine, Royal College of Surgeons in Ireland, D02 YN77 Dublin, Ireland; 2 Nuffield Laboratory of Ophthalmology, Department of Clinical Neurosciences, University of Oxford, Oxford OX3 9DU, UK; 3 Department of Ophthalmology, Mater Misericordiae University Hospital, Eccles Street, D07 R2WY Dublin, Ireland; 4 UCD School of Medicine, University College Dublin, Belfield, D04 V1W8 Dublin, Ireland; 5 LEP Biomedical Ltd., Nás na Ríogh, W91 HYH9 Cill Dara, Ireland

**Keywords:** glaucoma, rabbit, glaucoma filtration surgery, experimental design, pre-clinical, trabeculectomy, glaucoma drainage device

## Abstract

**Background**: The rabbit (*Oryctolagus cuniculus*) is a currently indispensable pre-clinical model for fundamental discovery and translational research in glaucoma surgery. While not fully representative of the human eye, the rabbit often serves as the site of the first robust assessment of a new chemical entity in an anatomically and physiologically relevant setting. In later stages of development, the rabbit is also a critical model for regulatory-facing Good Laboratory Practice (GLP)-graded pre-clinical safety studies. However, while guidelines exist for human glaucoma surgeries in terms of patient profile vs. surgery type and success criteria, there is limited collated information available on how rabbit glaucoma filtration surgeries are designed, use and mode of administration of anti-metabolite Mitomycin-C (MMC) and post-operative antibiotic and anti-inflammatory dosing regimes. These disparities limit cross-comparability in studies and encourage sub-optimal study design and incomplete reporting. **Methods**: This review investigated and collated current procedural and reporting practices based on 100 peer-reviewed rabbit glaucoma filtration surgery publications from the years 2011–2025. Publications meeting acceptance criteria were segmented into pre-operative designs (ethical approval, rabbit characteristics, study duration), intra-operative (surgery type, surgical approach, use of MMC) and post-operative follow-up (antibiotics, anti-inflammatories, analysis and adverse events). **Results**: It was found that there were clear preferences in animal model selection, study design and intra-/post-operative anti-fibrotic/inflammatory treatment regimes. Additionally, there were several areas identified where reporting was ambiguous or omitted, e.g., rabbit age, post-operative treatments, adverse events. The levels of omission were 16–30% and represent areas for improvement and highlight the need for ongoing review in pre-clinical research.

## 1. Introduction

Glaucoma is the leading cause of irreversible blindness worldwide with a prevalence of 76 million patients in 2020 [1]. In the USA, the estimated annual medical cost of glaucoma amounts to an additional $1863 individually and $9.2 billion nationwide [2]. In the European Union, a 2005 study estimated the additional economic burden per patient to be €455 per person year for stage 0 (low severity) to €969 per person year for stage 4 glaucoma (high severity) [3]. In the more than twenty years since this study was published, these costs have likely become higher still.

On diagnosis, especially that of Ocular Hypertension (OHT) or Primary Open-Angle Glaucoma (POAG), current first-line approaches to treatment focus on modulation of IOP via medical or laser-based interventions [4]. In cases where a clinically significant reduction in IOP is unattainable even at the upper limit of drug interventions and laser therapy, incisional surgical procedures are indicated for the management of glaucoma [5,6]. In recent years, these procedures have been broadly categorised into Minimally Invasive Glaucoma Surgeries (MIGSs), Minimally Invasive Bleb-forming Surgeries (MIBSs) and more traditional trabeculectomy surgeries and glaucoma drainage device (GDD) implants [7]. For the purposes of this review, all these later-stage surgeries (MIBSs, trabeculectomy and GDDs) have been broadly categorised as glaucoma filtration surgeries (GFSs).

The use of GFS is guided by the patient profile and the specific outcomes desired. Typically, MIGSs function best in mild–moderate glaucoma or glaucoma–cataract patients that have accumulated a high IOP-lowering drop burden where a modest decrease in IOP is required [8]. These surgeries are characterised by minimal manipulation of the ocular surface and conjunctiva, the lack of a filtering bleb and rare use of anti-metabolites such as Mitomycin-C (MMC) to ensure successful outcomes.

In contrast, GFSs—defined as MIBSs, trabeculectomies and GDDs—are most often used in patients whose visual field is deteriorating, who are unresponsive to prior medical, laser and/or MIGS interventions and who require double-digit decreases in IOP [6,7]. In these surgeries, use of MMC to ensure successful outcomes is standard practice (less so for GDDs) [9,10]. Trabeculectomy is the most performed glaucoma drainage surgery worldwide but use of MIBSs and tube shunts are widespread globally [11].

While trabeculectomy and tube surgeries are well established and widespread in use, innovation is constant in glaucoma drainage surgery. Most often, research focuses on the development of new surgical devices or pharmaceutical adjuvants to ameliorate post-operative inflammation, excess fibrosis and conjunctival scarring [12,13].

Pre-clinical assessment via animal models represents a critical milestone in the comprehensive validation of any emerging medical device and/or pharmaceutical compound. Wide varieties of animal models of different species have been used for glaucoma surgery research [13,14]. These include mice, rats, rabbits, dogs, pigs and non-human primates. Mice and rats offer advantages in terms of cost, availability for large group sizes and genetic knock-out models. However, the size of and anatomical differences in rodent eyes make these animals unsuitable for many device-orientated research projects in particular. Larger animals such as dogs, pigs and non-human primates offer greater levels of fidelity to the human patient but are often cost-prohibitive for statistically robust studies with no prior in vivo validation. Early use of large animal models also presents ethical constraints when success of early-stage research remains relatively uncertain.

Current guidance from the US FDA regarding pre-clinical model selection follows the “least burdensome” concept. Specifically, “the minimum amount of information necessary to adequately address a relevant regulatory question or issue through the most efficient manner at the right time” [15]. In the case of pre-clinical studies, “the selected animal model should be able to address the identified objectives of the study” [16].

As a pre-clinical model for GFS, rabbits offer a strong trade-off between anatomical and physiological relevance, husbandry, budgetary and ethical considerations. With an average horizontal corneal diameter of 11.75 mm, which closely approximates the human eye, clinical trabeculectomy and tube surgeries can be closely modelled in experimental rabbit models [14,17]. Furthermore, rabbits are prolific in their post-GFS inflammatory and fibrotic response. This therefore allows for a robust and cost-effective assessment of any new approach to modulation of the post-operative inflammatory/fibrotic cascade.

However, while guidelines exist for human studies [6] and consensus documents have been published for ex vivo experiments [18], there are limited resources available that standardise this critical pre-clinical model despite its significant role in the development of novel GFS modalities. Furthermore, reporting on anti-fibrotic and anti-inflammatory drug administration from different research groups is similarly heterogeneous. These issues place avoidable obstacles to optimising the design of future studies and can create confounding variables.

Prior glaucoma pre-clinical reviews have focused more generally on the advantages and disadvantages of various animal models in GFS [13,14,19] but have not collated any common practices in pre-clinical model design or preferences for dose and route of administration for MMC, antibiotics and anti-inflammatory drugs. As such, these issues need to be addressed to ensure higher reproducibility and reliability to discern at an early stage what can be translated to human patients.

This review aims to investigate current model design, intra-operative and post-operative drug-treatment regimes and reporting practices in GFS rabbit pre-clinical models. From this, it is hoped that an initial identification of common practices in rabbit GFS models may contribute to the development of more standard practices and the creation of a more widely comparable system for future studies. An additional secondary aim from this review is the identification of any gaps or areas for improvement in the post hoc reporting of rabbit GFS in the scientific literature.

## 2. Materials and Methods

The research articles were retrieved by using PubMed only (www.ncbi.nlm.nih.gov/pubmed/, accessed on 4 March 2026). Briefly, the following search terms were defined: “trabeculectomy”, “glaucoma drainage devices”, “glaucoma filtration surgery”, “rabbits”. A full description of the search strategy is available here:

(((((trabeculectomy) OR (“glaucoma filtration surgery”)) OR (“glaucoma drainage device” OR “glaucoma drainage devices” OR “glaucoma drainage implants”[MeSH Terms])) OR (“glaucoma implant surgery”)) AND (glaucoma)) AND (“rabbits”[MeSH Terms] OR “rabbits”[All Fields] OR “rabbit”[All Fields] OR “rabbit model”[All Fields]).

On receiving each search report, the inclusion and exclusion criteria described in Table 1 were applied to each paper. Appropriateness of each paper was assessed by MK, AAC, LB, ML, TA, GS, and AH with a minimum of two reviewers independently evaluating each article. Eligible publications were then read, and relevant data was extracted manually by the reviewers. A full list of reviewed, accepted and discarded papers is available in the Supplementary Material (Supplementary Table S1).

Extracted data was collated and stored using Microsoft 365 Excel 16 and Access 16 software. Additional statistical analysis formatting where required was undertaken using GraphPad Prism V11. Mode of anaesthesia was not considered as a metric as this is typically the responsibility of the designated veterinarian (DV) [20]. As such, it is not a variable under the control of the investigator.

Given the diffuse nature of pre-clinical research and the variety of experimental technologies for each study, meta-analysis that would assess correlation between any specific experimental design and successful surgical outcomes was not attempted.

## 3. Results and Discussion

### 3.1. Data Sourcing and Ethical Compliance

**Figure 1:**
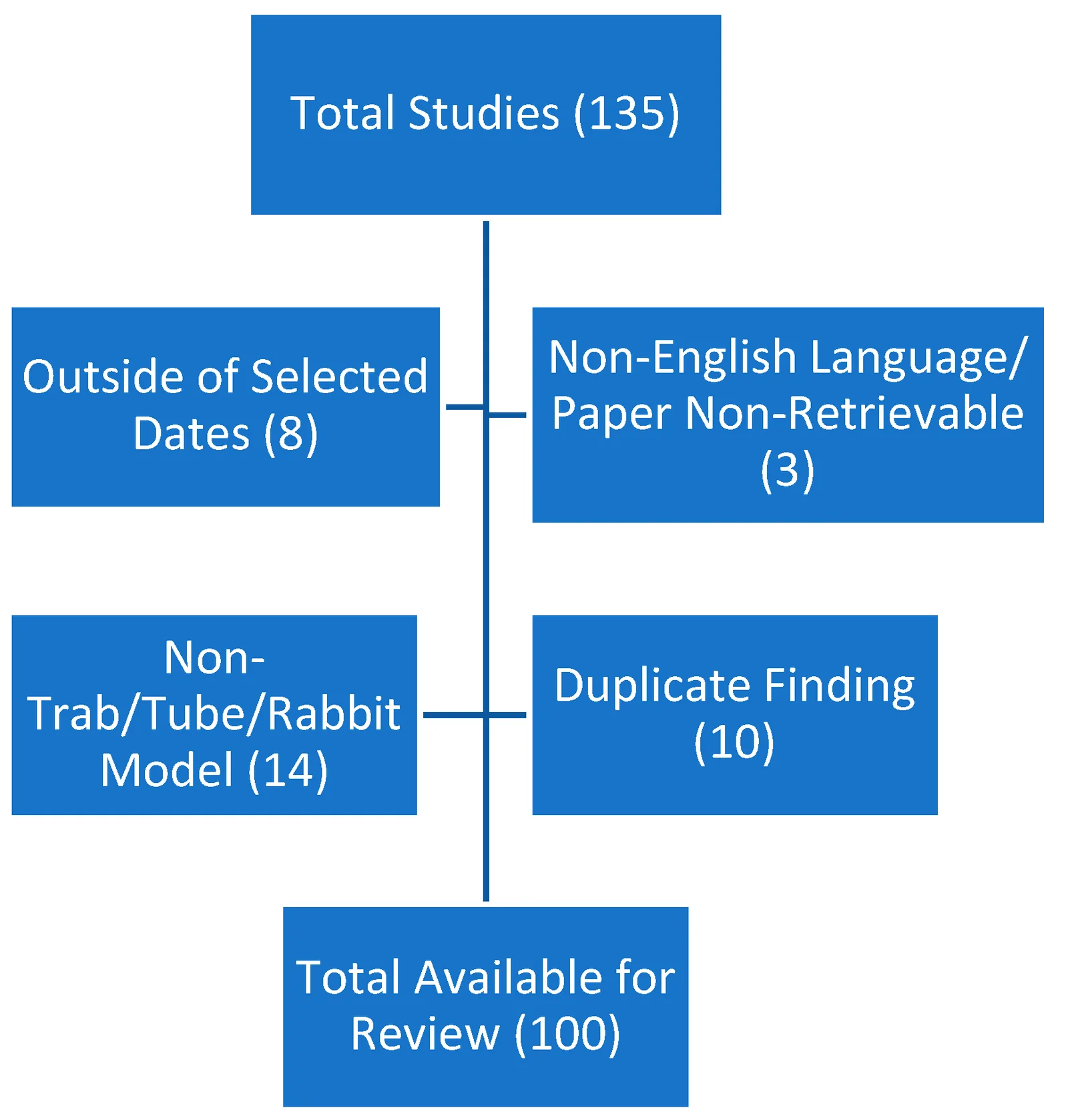
Selection and attrition process for reviewed papers within this study.

Following the described search and evaluation criteria, an initial total of 135 publications were identified (Figure 1). Of these papers, eight were discarded due to publication dates outside of the specified range, three were discarded due to the lack of any available English language version, a further fourteen were discarded due to nonrelevant surgeries or the lack of a drainage surgery of any type and ten publications were identified as duplicated and removed. This left a final total reviewable figure of 100 peer-reviewed articles.

**Figure 2:**
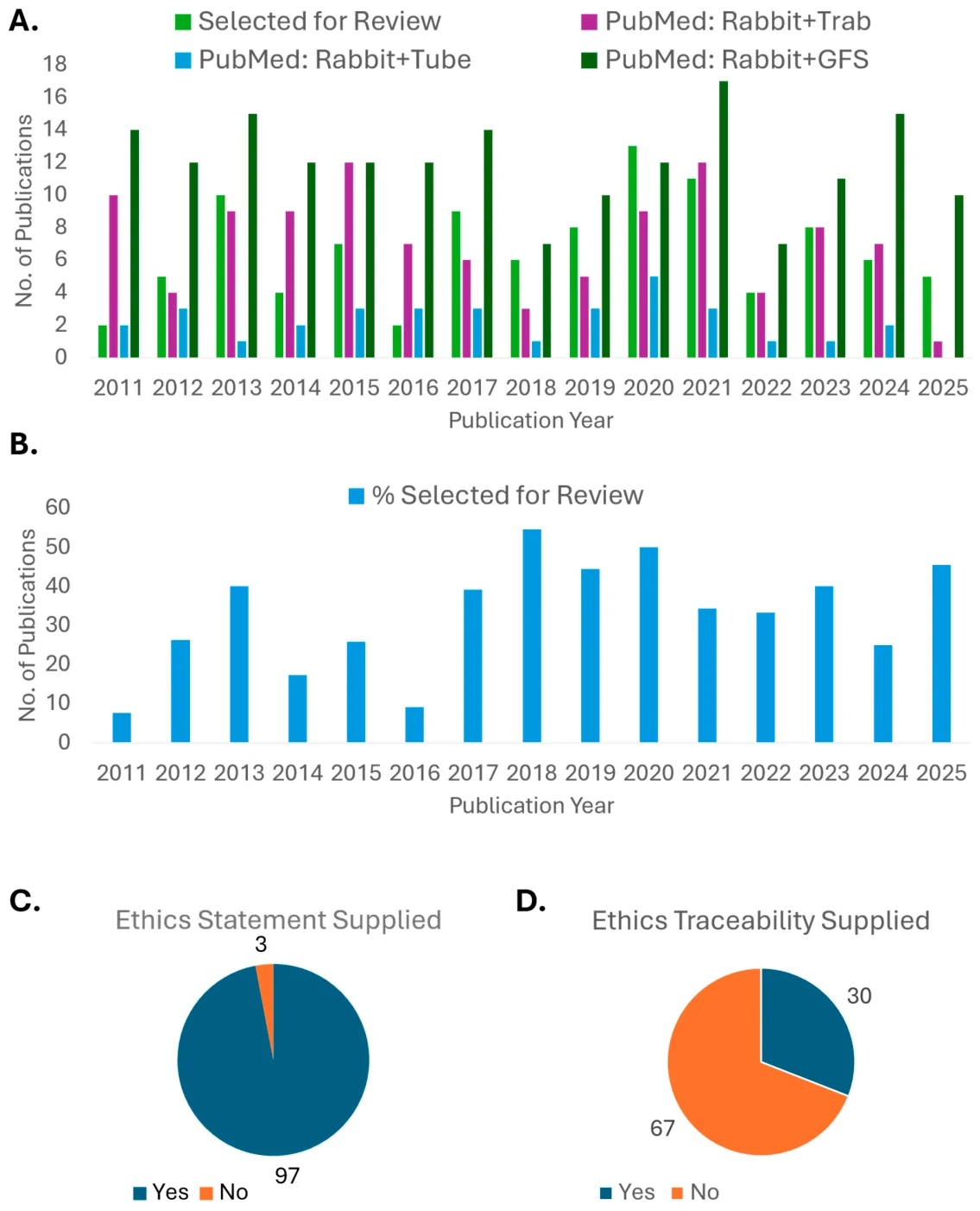
(**A**) Comparison of the number of selected papers vs. the total number of published papers by year according to relevant PubMed searches. (**B**) Comparison of selected papers as a percentage of total available papers 2011–2025. (**C**) In total, 97% of peer-reviewed papers supplied some form of statement of ethical compliance. (**D**) Where an ethical statement was supplied, 67/97 peer-reviewed papers did not supply any additional traceability information on their ethical approval.

To establish whether the one hundred papers were a sufficiently representative population of the larger rabbit pre-clinical GFS publication landscape, the number of selected papers was compared by year against the total number of PubMed results for “Rabbit+Trabeculectomy”, “Rabbit+Glaucoma Drainage Tube” and “Rabbit+Glaucoma Filtration Surgery” (Figure 2A). The number of publications selected for review in a given year varied from two (2) (years 2011/2016) up to a maximum of fifteen (15) (year 2020).

Selected publications were further compared as a percentage of the combined returned number of publications from all three search criteria (Figure 2B). Here, the average percentage of selected papers from the total available was 32.84 ± 14.11% across the years 2011–2025. When the two low-yield years of 2011 and 2016 are excluded, the average percentage of selected papers from total available rises to 36.63 ± 14.11%.

Following this, selected papers were assessed for compliance with ethical standards in animal use. It was found that there was a high level of stated compliance (97%) with either ethical review or application of the ARVO guidelines for animal use [21] (Figure 2C). However, when examined further for studies that supplied a traceability indicator for their ethical review (i.e., a case/application number), there was a large downward shift in compliance, with only 30/97 publications providing this information (Figure 2D). While it is debatable whether this detail should be seen as an absolute requirement, the lack of any traceability metric essentially closes the study in question to outside oversight.

### 3.2. Rabbit Characteristics at Study Launch

**Figure 3:**
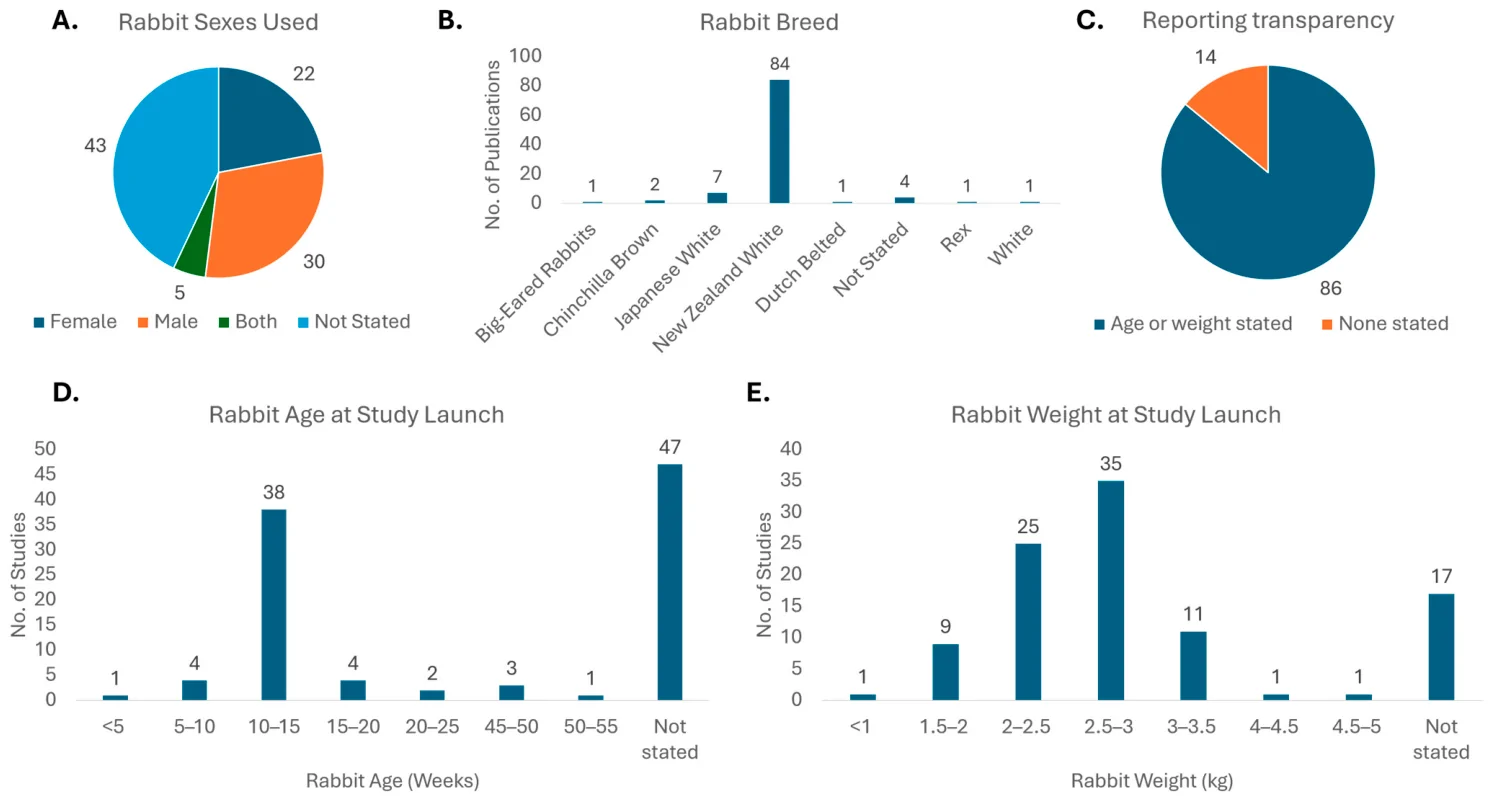
Rabbit selection and characteristics prior to study launch. (**A**) Reporting of rabbit sex. (**B**) Breed of rabbit selected for GFS study; 101 animal types are reported as one study used both NZ White and Dutch Belted. (**C**) Reporting transparency across 100 studies for providing either age or weight of rabbits in study design. (**D**) Reporting practices for rabbit age in weeks. (**E**) Reporting practices patterns for rabbit weight in kg.

Reporting of key biological characteristics of rabbit models prior to study launch demonstrated considerable heterogeneity. Sex of the animals used was documented in only 57% of all studies. Within the reported studies, 30 were male-only, 22 were female-only and 5 were mixed male and female (Figure 3A).

In recent years, there has been an increase in appreciation of the risks of single-sex pre-clinical and clinical studies and the subsequent creation of blind spots in later broader populations [22,23,24,25]. This was specifically addressed in the USA by the National Institute for Health in a 2015 Guide notice (NOT-OD-15-102) that highlighted the expectation of the NIH that the possible role of sex as a biologic variable be factored into research design, analyses, and reporting in vertebrate animal and human studies [26].

For GFS, sex-based hormones have a known effect on vasodilation/constriction, with oestrogen exerting a vasodilatory effect and testosterone being associated with vasoconstriction. This may have implications for uveo-scleral outflow and the overall IOP-lowering effects of GFS interventions. Similarly, inflammation and scarring processes have been found to exhibit sex-based differences on a molecular level in dermal studies [27].

However, from the small population of male and female rabbit studies identified, there were no differences in outcomes noted. Furthermore, numerous human clinical studies have not identified any sex-based variation in GFS success [28].

It must also be acknowledged that the use of mixed-sex populations in rabbits can present challenges in terms of appropriate housing. It may also be cost-prohibitive or impractical in terms of timelines to assess new GFS outcomes in different sexes concurrently. However, there are few reasons to omit reporting this characteristic from any pre-clinical publication.

In terms of selection of the specific breed/strain of rabbit used, there was a large preference (84%) for the use of the New Zealand White rabbit (Figure 3B). This reflects their historical prominence in ophthalmic surgical modelling given their widespread availability and well-established characteristics. Smaller contributions were recorded from Japanese White, Albino, Chinchilla Brown, White, Rex, and Dutch Belted strains. Additionally, four studies did not provide any breed/strain information.

Assessment of the reporting of rabbit age or weight (as an indicator of maturity) found that 14% of studies reported neither (Figure 3C). This is an obvious area for improvement as the lack of this information limits the reader from fully contextualising the outcomes described.

Of the studies examined in this review that reported rabbit maturity markers via age (Figure 3D) or weight (Figure 3E), there were levels of clustering noted. Where rabbit ages were recorded, the clear preference for animals was between 10 and 15 weeks of age (69% of reported ages). Rabbit ages ranged from approximately 3.5 to over 50 weeks old, and reporting was incomplete, with 47% of studies not providing a numeric age.

Mean body weight was reported more consistently (83% of studies), but with greater variation. Described weights spanned 0.3–5.5 kg with 35% of reported animals weighing between 2.5 and 3 kg. This corresponds to an age of 12–16 weeks for New Zealand White Rabbits according to [29]. It is also worth noting that an additional 34% of reported animals were below this weight range and could be considered juveniles.

Depending on the desired goals of the study, such young animals may potentially skew the safety and efficacy outputs of any study.

There is a demonstrated relationship between animal age/sexual maturity, IOP and aqueous humour dynamics. Juvenile (approx. three months old) New Zealand White rabbits demonstrated significantly lower IOP and significantly higher rates of anterior chamber volume, aqueous flow and uveo-scleral outflow compared against adult (approx. 10–12 months old) New Zealand White rabbits [30,31]. Similarly, in Dutch Belted rabbits, IOP was found to increase significantly from 10 to 18 weeks. In the same study, anterior chamber depth was also found to increase significantly over the same time period [32]. All of these characteristics contribute to the eventual outcome of the surgery.

Any evaluation of IOP reduction or management of IOP reduction (for anti-scarring studies) may be compared against a starting IOP, contra-lateral un-operated IOP or control-treatment-group animals. Unintentionally low starting IOPs in rabbits risks false negatives in the first two controls described and false positives in the latter group.

While the purpose of pre-clinical studies in regulatory facing studies is safety, this is not the case in earlier, discovery-stage research, and sub-optimal rabbit selection may inadvertently terminate an otherwise promising project.

Similarly, unrealistic safety data may be returned due to sub-optimal animal characteristics, for example, the problematic insertion of an otherwise safe drainage tube due to the fact the anterior chamber was at a depth that is not clinically relevant. Possible risks include: unnecessarily high levels of corneal endothelial cell loss due to a low anterior chamber and tube–endothelial touching, device displacement through animal growth, and early occlusion owing to an excessively robust recovery due to a juvenile rabbit’s physiology. As previously mentioned by Gulati et al. [32], anterior chamber depth in Dutch Belted rabbits rose significantly from 1.93 ± 0.25 mm at week 10 to 2.46 ± 0.09 mm at week 18. In contrast, human anterior chamber depths vary by geography: e.g., 2.42 ± 0.34 mm in the Beijing Eye Study [33], 2.92 ± 0.44 mm in a Northern Mexican (Monterrey) Study [34] and 3.15 ± 0.24 mm in healthy and 3.12 ± 0.27 mm in POAG patients in a study based in Ile-Ife, Nigeria [35].

Based on the maturity data described in Figure 3D,E, the majority of rabbits used were not of an age likely to approximate the lower end of human anterior chamber depths. This may therefore also be an unfortunate contributing factor to a hypothetical project failing to meet its desired endpoint.

It is worth addressing the counterpoint that the rabbits as currently chosen are acceptable given the positive findings so often described. This assumption fails to address the well-established bias towards publication of positive results only [36,37,38,39]. An additional counterpoint may be that the use of visco-elastics or balanced saline solution injected into the anterior chamber during surgery may offset some safety risks posed by smaller chamber depths. While this is common practice, any inflation of the anterior chamber is temporary, and the risk may return as drainage increases. Furthermore, as noted in Section 3.7 of this review, rabbit GFS studies very often do not adequately describe adverse events in their results sections (discussed under “Morbidity and Mortality Reporting”). As such robust correlations were not possible at this point, these items are flagged for future consideration for any researcher considering a rabbit GFS study.

There is also the issue that it could be cost-prohibitive or impractical from a housing perspective to move to older animals. However, the cost and housing considerations that may allow a higher sample size of younger animals are irrelevant if the data returned is not reflective of the disease state, especially when otherwise promising technologies are abandoned due to false negatives.

Overall, selected studies were highly concentrated in terms of rabbit breed and age. This represents a highly homogenous test population and therefore offers a considerable opportunity to establish a more harmonised approach in all subsequent activities described herein.

### 3.3. Glaucoma Filtration Surgery Technique

**Figure 4:**
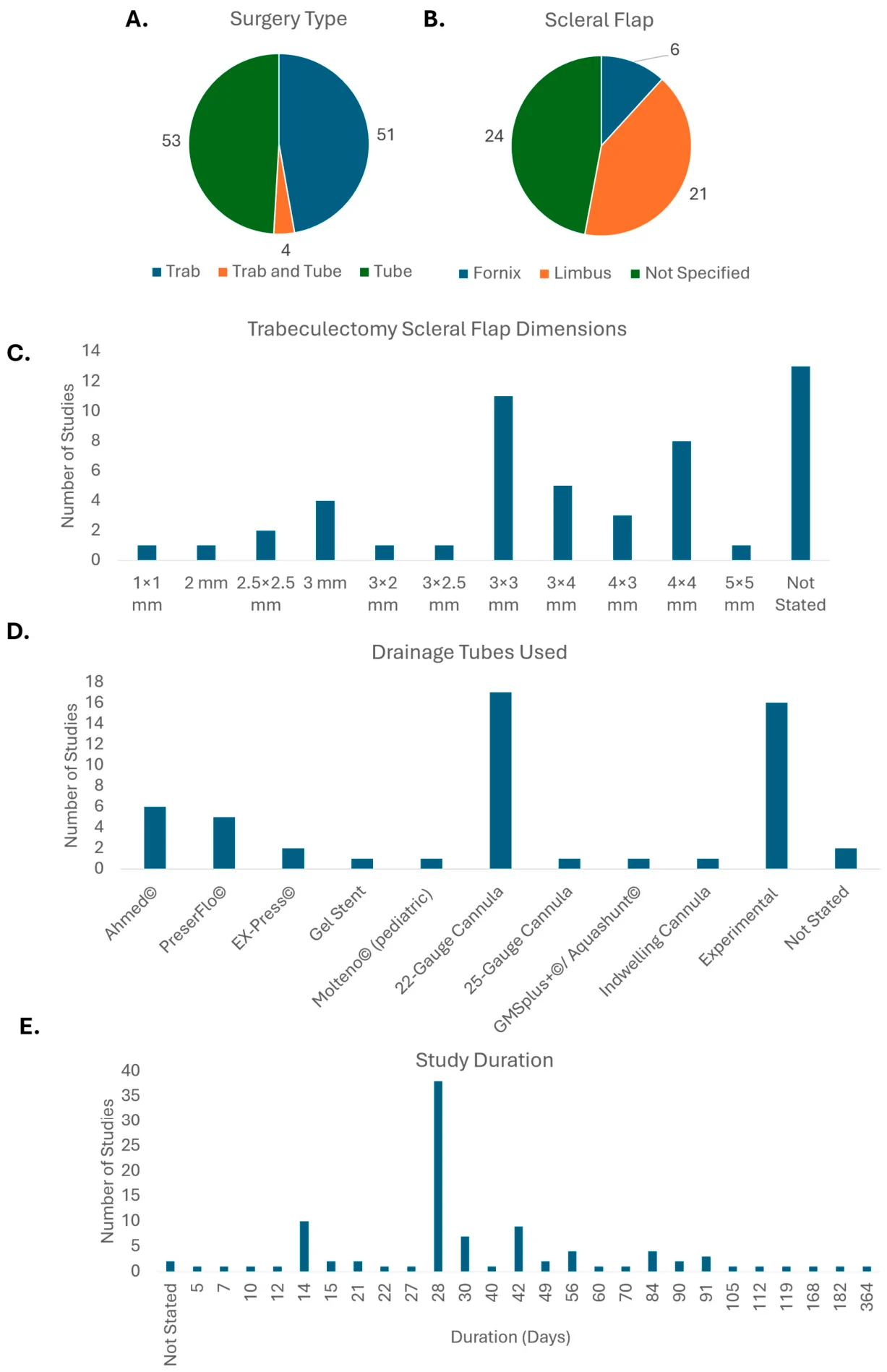
(**A**) Surgeries were categorised as either trabeculectomy (“Trab”) (51), glaucoma drainage tube (“Tube”) (53) or both (4). (**B**) Scleral flap creation preferences in the 51 rabbit trabeculectomy studies examined. (**C**) Scleral flap dimensions. (**D**) Overview of tubes used for reductions in IOP. (**E**) Overall study duration by number of days post-surgery.

Of the studies reviewed, there was a distribution of 51 publications that utilised trabeculectomy as the primary surgery and 53 that utilised tubes of some manner to lower IOP. Additionally, four studies utilised both trabeculectomies and tubes (Figure 4A).

Within the trabeculectomy studies, 41% of studies favoured use of a limbus-based scleral flap with 12% utilising a fornix-based approach (Figure 4B). However, the surgical approach was frequently under-reported with 47% of studies making no mention of the direction of approach for scleral flap creation. This lack of reporting is notable given the well-established differences between fornix- and limbus-based techniques in terms of wound healing, bleb morphology, and complication profiles in human surgery [40,41].

Additionally, there was a considerable amount of variation in the dimensions of the scleral flaps created (Figure 4C). A further 26% of trabeculectomy studies did not report the dimensions of the scleral flap created, while 47% of trabeculectomy studies created a flap where at least one of the dimensions was 3 mm and 21.5% of all relevant studies utilised a 3 × 3 mm scleral flap. Following this, 31% of all trabeculectomy studies utilised a scleral flap with at least one dimension being 4 mm and 16% of all trabeculectomy studies were 4 × 4 mm. Taken together, there is an evident concentration of a surgeon preference between 3 and 4 mm for scleral flap creation.

Within the glaucoma drainage tube studies, a similarly wide range of devices and cannula types were reported (Figure 4D). As might be expected from early-stage research, 30% of studies reviewed were classed as using “experimental” drainage tubes, i.e., those made in-house by the study authors and not a third party.

Of the commercially available tubes, the most common approach was the use of a 22-gauge cannula to create a drainage aperture in the rabbit eye (32% of tube studies). There were also a further two studies utilising other cannula-based approaches. This increased the frequency of use for cannula-based approaches to 36% across the total set of tube studies. The use of a cannula (primarily the Insyte^®^ by Becton Dickinson (Franklin Lakes, NJ, USA)) represents a pre-clinical-model-specific approach to creating a decrease in IOP with greater reliability than might be achieved with a standard trabeculectomy. This approach was most often described as “glaucoma filtration surgery” rather than any specific surgery. However, it was also referred to as a “modified trabeculectomy” in one study and incorrectly as trabeculectomy in a further two studies.

For clinically approved tubes, the Ahmed^®^ Glaucoma Valve (New World Medical, Rancho Cucamonga, CA, USA), PreserFlo^®^/SIBS microshunt (Santen, Osaka, Japan), EX-PRESS^®^ device (Alcon, Fort Worth, TX, USA), XEN^®^45 gel stent (AbbVie, North Chicago, IL, USA), Baerveldt^®^ glaucoma implant (Johnson & Johnson Vision, New Brunswick, NJ, USA), paediatric Molteno^®^ implant (Nova Eye Medical, Adelaide, Australia), polymethyl methacrylate GDD (Rohto Corp., Jakarta, Indonesia), nitinol microstent from Ivantis (now acquired and rebranded by Alcon), polypropylene shunt (OPKO Health, Miami, FL, USA) and gold shunts (GMSplus, SOLX Ltd., Waltham, MA, USA) all featured. Two additional studies did not report the origin of the drainage tubes used.

Following conclusion of the primary surgery, duration of the study was assessed across all surgery types (Figure 4E). The duration of follow-up varied widely across the included studies. Follow-up periods ranged from as short as 3 days to as long as 364 days. This reflects the varied nature of pre-clinical research and the individual aims of each study.

Of the durations examined, 38 studies had post-op follow-up durations of 28 days, followed by 14 days (10 studies) and 42 days (9 studies). Only two studies failed to report the duration of post-op follow-up.

While studies will vary depending on specific aims, it would be hoped that sufficient time is given for studies to accurately account for the full completion of the wound healing response. Given the inflammatory response lasts approximately 7 days and the proliferation and tissue remodelling response in wound healing is established and ongoing at 4 weeks, the preference towards 28-day study durations is appropriate as a minimum duration for studies focussed on wound repair [42].

### 3.4. Mitomycin-C Administration and Dose

Mitomycin-C (MMC) has been an integral part of clinical glaucoma drainage surgeries since its introduction in 1983 [43]. As an anti-metabolite, MMC is primarily used as an anti-tumour agent. MMC functions as an alkylating agent that inhibits DNA synthesis primarily during the late G1 and S phases [44]. In glaucoma, MMC is applied to decrease the risk of post-operative complications arising from excessive conjunctival inflammation and fibrosis (scarring).

Anti-metabolite use in the UK in 2004 was 82% in clinical trabeculectomy [45]. MMC use specifically is now standard of care given its use in 97% of primary trabeculectomies according to a 2017 American Glaucoma Society survey [10]. MMC is also commonly used in sub-conjunctival microshunts such as the Xen or PreserFlo [46,47,48]. MMC use in GDDs such as the Ahmed Glaucoma Valve tubes is less common due to the questionable impact of MMC; however, their combined use is under investigation [6,49,50].

**Figure 5:**
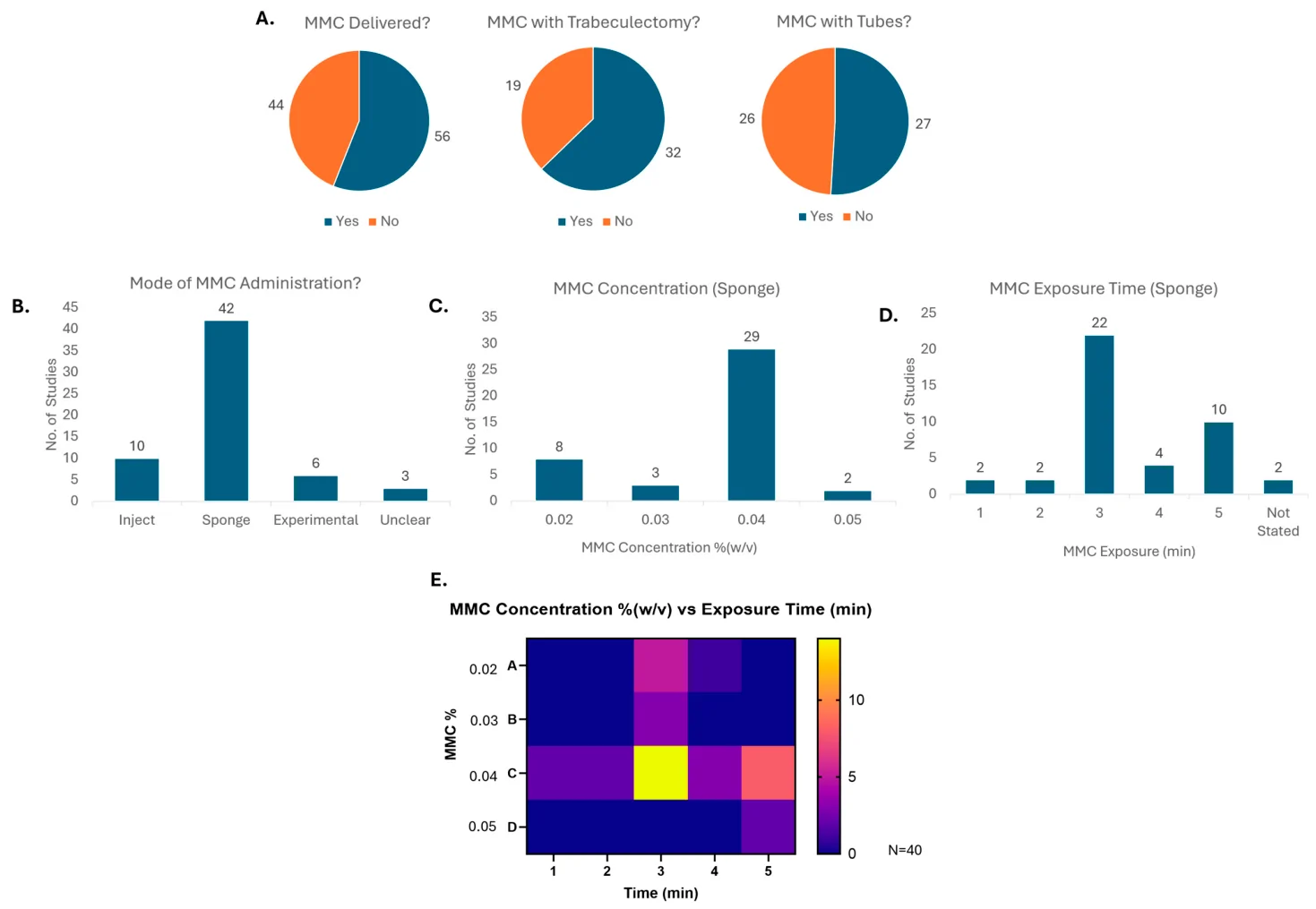
(**A**) Use of MMC by number of studies: (**left**) overall (four studies used two types of administration), (**middle**) in trabeculectomy and (**right**) in tube-based rabbit pre-clinical studies. (**B**) MMC use by method of application. (**C**) Concentration of MMC when applied by sponges/swabs. (**D**) Exposure duration of MMC when applied by sponges/swabs. (**E**) Heatmap of MMC concentration vs. exposure time in sponge/swab use with frequency of use by publication on the righthand *y*-axis.

In contrast, use of MMC was considerably lower in rabbit pre-clinical models (Figure 5A). Overall use across all studies was 56% with 61 incidents of MMC application, i.e., four studies contained multiple MMC application approaches (sponge vs. experimental, sponge vs. injection, etc.). MMC use in trabeculectomy-based studies was at 63% and use in tube-based studies at 51%. This lower than clinical level of MMC use is likely a reflection of the “first look” nature of pre-clinical testing. Assuming no safety concerns were discovered in prior in vitro work, it is reasonable to assess the effect of any new technology in a standalone fashion. However, the majority of studies still preferred to include MMC in some manner, especially given the rabbits’ prolific ability to repair ocular tissue [51].

Where MMC was applied, approx 69% of administrations were a topical application via a surgical sponge or swab (Figure 5B). The remaining approaches were sub-conjunctival injection (16%) or experimental delivery methods (10%) and a further 5% of studies did not clearly communicate the means of MMC application, i.e., “MMC was applied”.

When applied with a sponge/swab, MMC was most often (69% of swabs) applied at a concentration of 0.04% *w*/*v* (Figure 5C). Duration of MMC exposure was more diffuse, with 3 min accounting for 52% of events, followed by 5 min in 24% of events. Approximately 5% of relevant studies did not define their exposure time (Figure 5D). MMC delivery by sponge was further stratified to match both time and exposure (Figure 5E). When combined, the most common MMC application remained 0.04% MMC for 3 min (35%). This was followed by 0.04% MMC for 5 min (20%) and 0.02% MMC for 3 min (12.5%). In comparison, the most commonly reported conditions for MMC use in human trabeculectomies were 0.04% applied for 4 min [10]. However, MMC use preferences in humans described by Vinod et al. were similarly diffuse, with the 0.04% MMC concentration only accounting for 48% of survey respondents and 4 min exposure accounting for 33% of respondents.

MMC use in clinical glaucoma cases remains mostly off-label. FDA-approved MMC for post-operative glaucoma surgeries exists in the form of Mitosol^®^ (now recently acquired by Glaukos, Aliso Viejo, CA, USA) [52] but it is not used extensively. In the absence of formal clinical approval, risk stratification and decision making have been left to the surgeon depending on the specific patient profile. For example, it is known that patients of Afro-Caribbean descent have a greater predisposition to post-operative scarring than those of Caucasian descent [28] and thus may potentially be treated more aggressively in terms of MMC dose and exposure time. According to the 6th Edition European Glaucoma Society (EGS) Guidelines, recommended MMC doses are 0.01–0.05% for 1–5 min for sponge-based administration [6].

While this flexibility in decision making for MMC use has a clear purpose at the clinical level, little to no population heterogeneity exists at the pre-clinical stage. As found in this review, there is a strong preference for New Zealand White rabbits, rabbit age and study duration. The possibility of a set dose of MMC and exposure time would allow for greater standardisation between independent research groups and eliminate waste in repeat experiments. This is most easily achieved in swab-administered MMC. It should not be taken that such a move would aim to eliminate the use of injected MMC at the pre-clinical level. This is especially the case when injected MMC is the most fitting comparator for any new anti-fibrotic drug, device or surgical technique.

### 3.5. Antibiotic and Corticosteroid Administration and Dosing

Management of infection and inflammation are crucial intra-operative and post-operative activities in pre-clinical and clinical settings. In clinical settings, post-operative infections (endophthalmitis) for glaucoma patients are rare but a significant concern [53]. Similarly, uncontrolled inflammation is the root cause of later collagen matrix deposition [42] and is a major contributor to surgical failure, needling interventions and repeat surgeries in trabeculectomies and sub-conjunctival shunts in particular [54,55].

**Figure 6:**
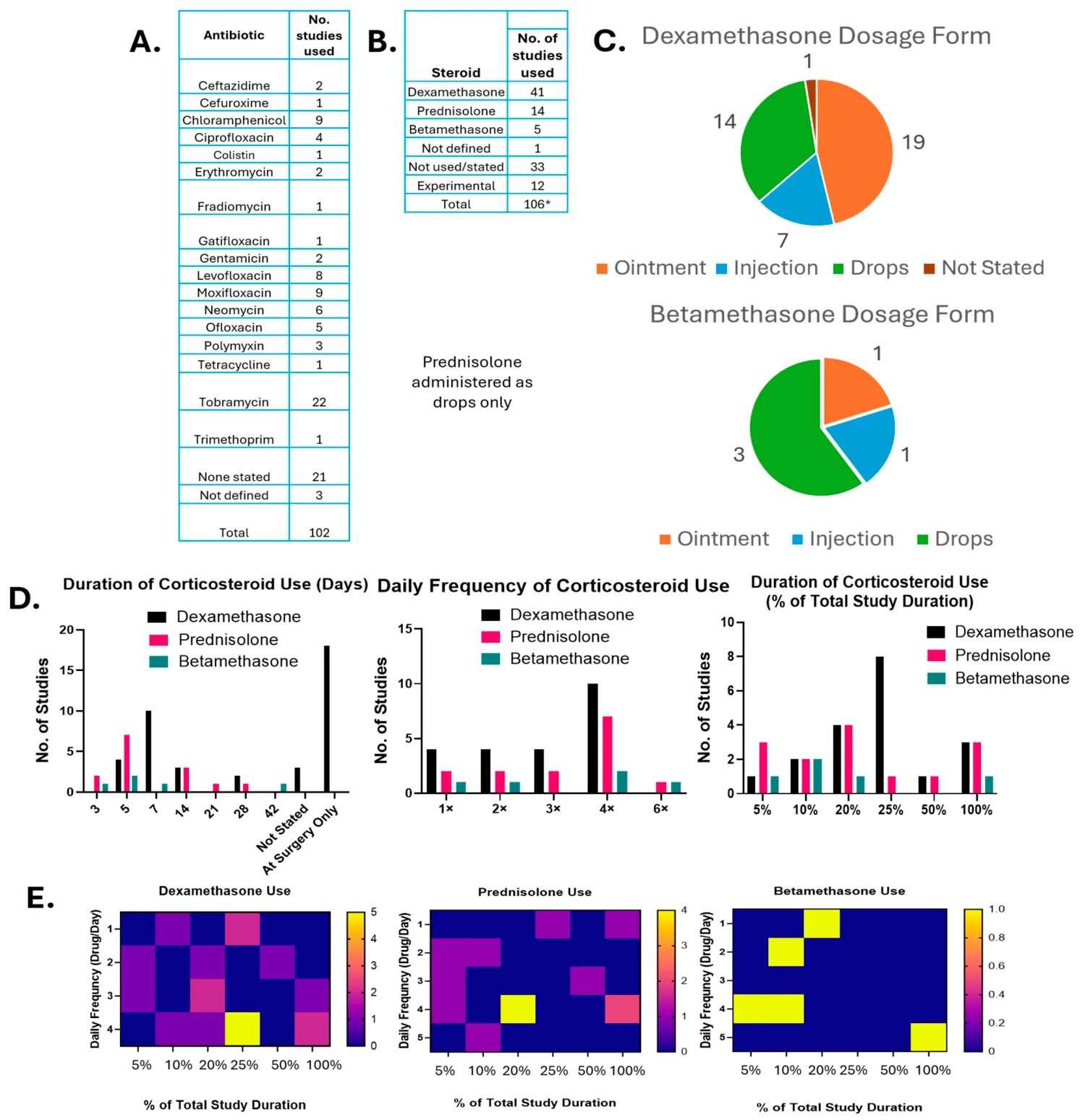
(**A**) Antibiotic use in rabbit pre-clinical GFS studies. (**B**) Corticosteroid/anti-inflammatory use in rabbit pre-clinical GFS. (**C**) Mode of administration for corticosteroids (n = number of studies). (**D**) Dosing regime of corticosteroids by daily frequency (**middle**), total duration of administration in days (**left**) and duration of administration as a percentage of total study duration (**right**). (**E**) Heatmap analysis of daily corticosteroid use vs. duration of administration as a percentage of total study duration (colour intensity indicates frequency of use by number of publications). * indicates use of multiple antibiotics/anti-inflammatories in some studies.

When antibiotic use was analysed for GFS rabbit models (Figure 6A), there was a 79.5% application rate in the reviewed studies. Three studies used multiple antibiotics to limit post-operative infections, and there were also three cases where the specific antibiotic was not clearly defined.

Of the clearly defined antibiotics used, tobramycin was the most commonly used (21.5% of studies) and was applied as dual tobramycin–dexamethasone formulation (Tobradex^®^ (Novartis, Basel, Switzerland)) in 20/22 of these studies. Beyond this, antibiotic use was variable, and no clear preference for use was identified.

The rate of reporting of anti-inflammatory use was 67% (Figure 6B). In total, 12% (12/100 studies) of anti-inflammatories were found to be experimental therapies and the remainder mostly comprised dexamethasone, prednisolone and betamethasone (one study did not specify the drug used). An overabundance of anti-inflammatories (106 instances of use vs. 100 publications) was also evident due to the fact there was one study which used three administrations of dexamethasone (drops, ointment, injections) and a further four studies that used two administrations of dexamethasone (drops and ointment (n = 2) and drops and injections (n = 2)).

The preferred means of delivery for dexamethasone (Figure 6C) was as a topical ointment (46.3% of dexamethasone applications (n = 19 studies)) followed by eye-drops (34.1%/ n = 14) and sub-conjunctival injections (17.1%/ n = 7), with 2.4%/n = 1 study not providing the specific means of administration. There was one instance of a dexamethasone dose (ointment) being tapered downwards, but no further details were given in this instance.

Betamethasone was delivered via eye-drops in three out of five studies, with the remaining use split between ointment and sub-conjunctival injections. There was one instance of a betamethasone dose (eye-drops) being tapered downwards over the duration of the rabbit study. As with dexamethasone, no additional details of the nature of dose tapering were provided.

Prednisolone was delivered using topical eye-drops in all cases. There were three studies that reported a tapering off prednisolone drops. In two of the studies, the dose was reduced by 50%. In the first of these studies, this was after week two post-op and the treatment was extended an additional two weeks (total study duration 84 days). In the second study, corticosteroid treatment was extended an additional 4 days to a total administration time of 7 days with a total study duration of 30 days. In the remaining study, prednisolone drops were reduced from six/day in week 1 to four/day in week 2 and stopped entirely after that point (total study duration 56 days).

In addition to the mode of delivery, there was also considerable heterogeneity in overall treatment regime for corticosteroids (Figure 6D). For dexamethasone-treated rabbits, of the 40 studies that specified a mode of administration, 18/40 delivered anti-inflammatories as a one-off at the end of surgery. A further three dexamethasone studies did not specify the duration of their treatment regime, and the remaining studies were clustered at the 5–7-day range (14/40 relevant studies). Neither prednisolone nor betamethasone were delivered as a “surgery only” treatment. Beyond that, a similar preference for delivery in the first week post-op was observed for prednisolone and betamethasone.

Treatment heterogeneity was also visible in the frequency of daily post-operative corticosteroid dosing. Of the twenty-two dexamethasone studies that were not “surgery only”, 10/22 studies dosed animals 4×/day and the remaining 12 studies were evenly distributed across 1×, 2× and 3× administrations per day. A similar pattern was observed for prednisolone and betamethasone.

When corticosteroid treatment regimes were assessed as a percentage of overall study duration, the majority of studies (73–80%) across all three corticosteroids concluded administration at 25% of the total study duration. (Figure 6E). Overall, there were clear preferences for high-frequency anti-inflammatory drug administration for the first 25% of a rabbit GFS study. This is not surprising given the acute and rapid nature of post-operative inflammation [42].

Finally, there were a considerable number of publications that did not report either antibiotic or anti-inflammatory treatments (21% and 33% respectively). In the case of anti-inflammatories, it is acknowledged that some researchers were looking to assess the initial foreign body reaction to a new technology or that the technology was devised to be anti-fibrotic (i.e., a surface coating). However, only 3/33 studies specifically highlighted that traditional anti-inflammatory care was omitted by design.

This scenario is much less likely to be the case for antibiotics, as sterility is a necessity. Considering the facility’s designated veterinarian would have final discretion to ensure animal wellbeing in most jurisdictions, there is a strong chance that researchers are not reporting use rather than the veterinarian opting to forego antibiotic or anti-inflammatory use entirely unless it formed part of the research question.

This lack of clarity limits full contextualisation of any results, especially in the case of emerging technologies. This is further compounded by the relatively large number of studies not reporting adverse events (discussed in Section 3.7).

### 3.6. Evaluation of Glaucoma Filtration Surgery Efficacy

**Figure 7:**
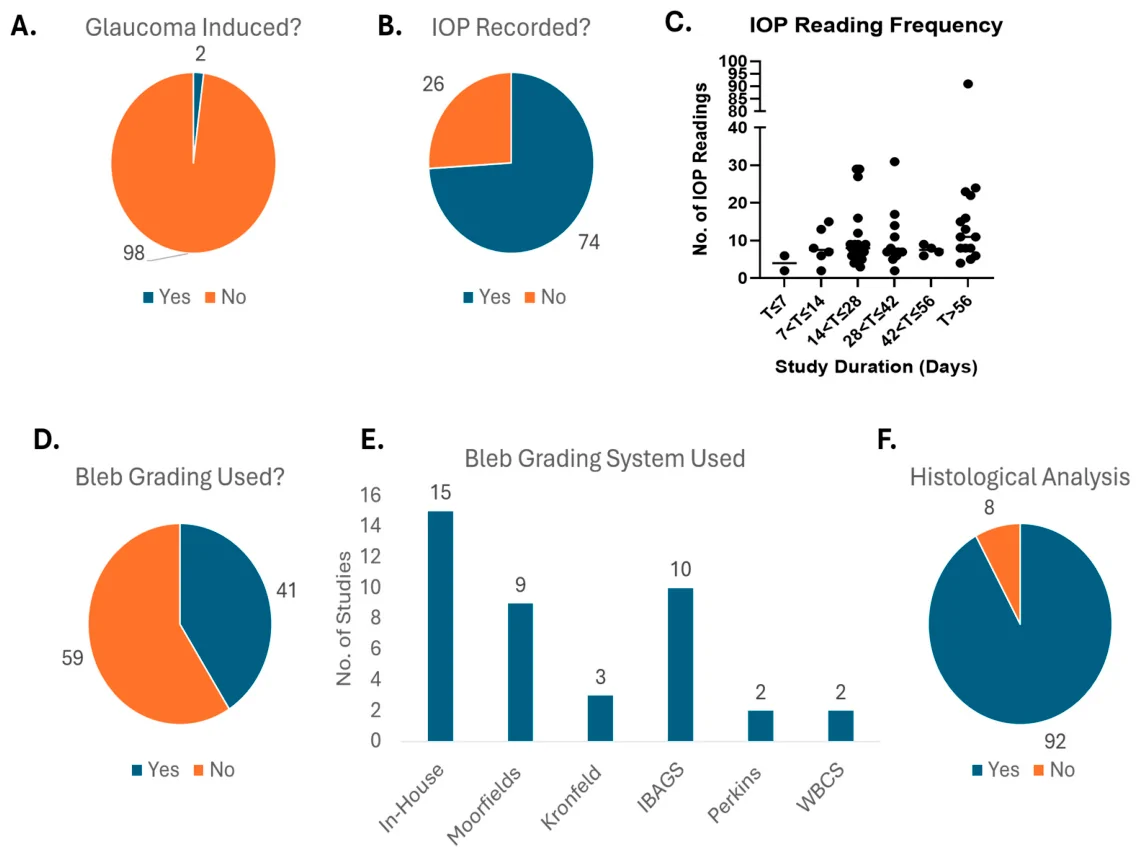
Assessment of outcomes in rabbit GFS models. (**A**) Induction of an ocular hypotensive/glaucoma state in the studies assessed. (**B**) Frequency of use of IOP as an assessment criteria. (**C**) Frequency of IOP measurements in rabbit GFS studies as a function of study duration. (**D**) Use of a bleb grading system in rabbit GFS studies. (**E**) Specific grading systems used. Abbreviations: Indiana Bleb Appearance Grading Scale (IBAGS), Wuerzburg Bleb Classification Score (WBCS). (**F**) Frequency of histological analysis as an assessment criteria in the rabbit GFS studies examined.

Evaluation of success criteria in GFS at the pre-clinical setting is more diffuse than in clinical settings. Human clinical glaucoma surgical interventions are usually appraised in terms of reduction in IOP-lowering drop burden, percentage decrease in IOP, visual acuity scores or OCT imaging [5,6]. However, given that 98% of rabbit GFS studies occurred in normotensive eyes (Figure 7A), combined with logistical and financial barriers, most of these assessments are not relevant.

In contrast, IOP assessments are easily performed in rabbits [56] and allow straightforward cross-referencing with clinical efficacy. Specifically, the same clinical cut-offs for success (i.e., a 20% reduction in IOP) can be applied to rabbit GFS [5].

Within the selected studies, the recording of IOP showed surprising variability. Overall use of IOP analysis was only described in 73% of publications (Figure 7B). This represents a considerable divergence from clinical practice where IOP change is a key metric of surgical success. This may simply reflect the more heterogenous nature of pre-clinical research. For example, the goals of some researchers may be discovery-focused rather than translational and thus be more focused on fundamental biological pathways rather than clinical markers.

However, for translational studies that choose to omit IOP readings, there is the possibility that important insights may be missed even if safety rather than efficacy is the initial goal. For example, corneal endothelial cell viability can change as a result of shear stress [57,58]. Therefore, safety data may be misleading depending on whether a tube is actively draining aqueous humour or has ceased to do so prematurely due to fibrotic encapsulation. Without IOP data, it may not be possible to fully contextualise such findings.

For the studies that did track IOP, the frequency of these readings varied considerably across different study durations, with no significant differences observed in IOP reading frequency across the different study durations (Figure 7C).

Heterogeneity in IOP examination was most pronounced at the extremes of study durations. For studies that were T > 56 days in duration (n = 15), the mean number of IOP examinations was 17.67± (±5.5 Standard Error of the Mean [SEM]), including one study reporting 91 individual IOP readings. In contrast, at the centre of the bell curve, readings numbered 9.9 ± 1.3 (SEM) and 10.17 ± 2.2 (SEM) for studies lasting 14 < T ≤ 28 (n = 32) and 28 < T ≤ 42 (n = 12) days respectively.

Visual inspection and grading of a filtering bleb were used in 59% of studies (Figure 7D) with a variety of grading systems used (Figure 7E). Internally devised or “in-house” grading systems were those that used a grading system that did not explicitly credit its origin. “In-house” systems accounted for 36.5% of reported systems, with known standards making up the balance. The most popular of these systems were the Indiana Bleb Appearance Grading Scale (IBAGS) and the Moorfields bleb grading system.

Post mortem evaluation was consistent across the literature; histological analysis was performed in 92% of the included studies (Figure 7F). This indicates its heavily established role as a standard metric for assessing wound healing, tissue tolerance, and fibrotic responses in rabbit pre-clinical models, with only 8% of studies omitting this type of analysis. However, as previously mentioned, there are some risks in relying on histological analysis when not paired with IOP readings.

Beyond IOP and histological analysis there were a variety of techniques used. However, these were too diffuse in nature and specific to the objectives of the individual study to allow for any meaningful conclusions to be made when pooled.

### 3.7. Morbidity and Mortality Reporting

**Figure 8:**
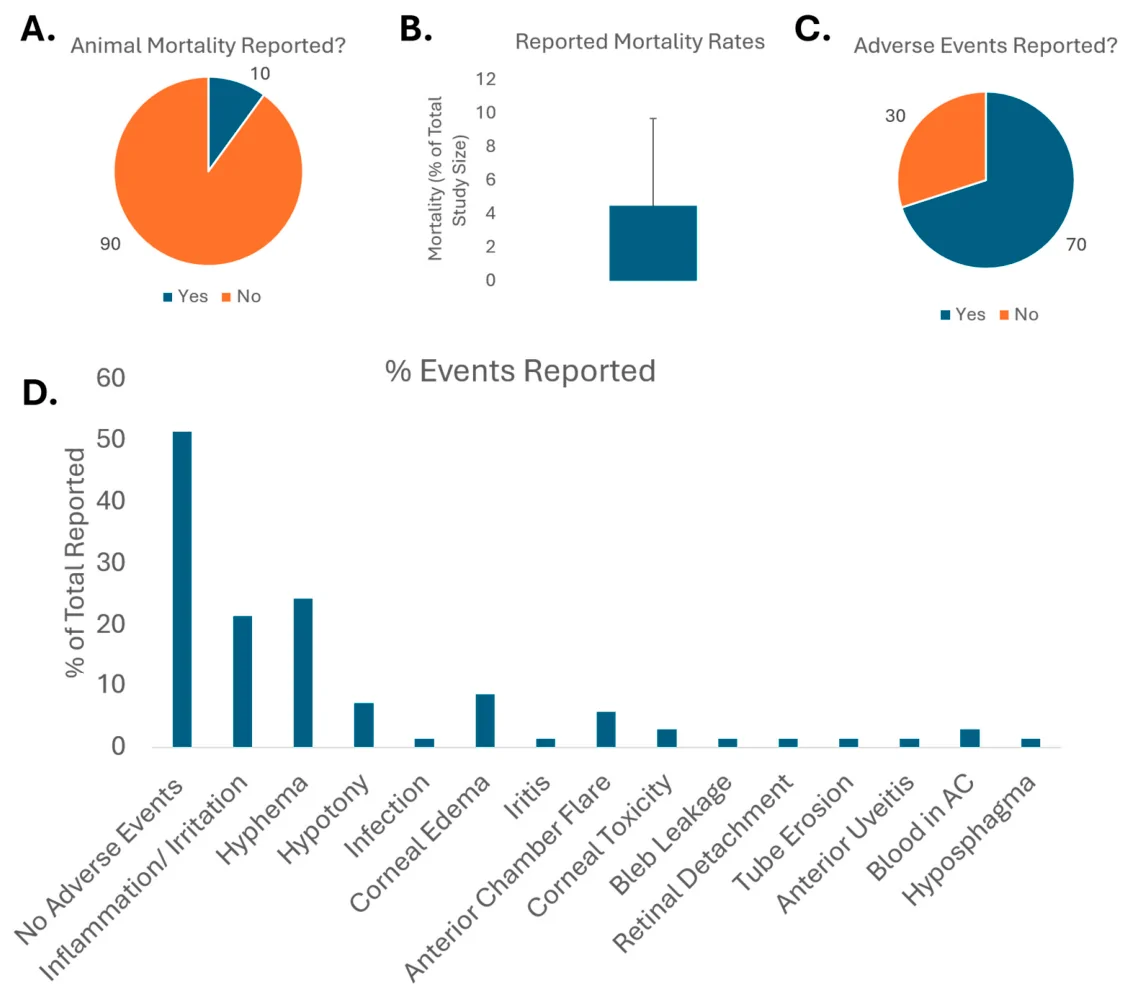
Morbidity and mortality reporting practices in rabbit GFS studies. (**A**) Reporting of mortality rates in rabbit GFS studies. (**B**) Mortality rates (where reported) as a percentage of starting population. (**C**) Reporting frequency of complications and adverse events in examined rabbit GFS studies. (**D**) Frequency of reported adverse events in rabbit GFS studies examined.

The reporting of animal mortality was found to be notably deficient across the reviewed literature. In total, 90% of the publications examined did not explicitly state whether any animal mortality occurred during the experimental period (Figure 8A). Of the 10% of studies that did track and report this metric, the mortality rate averaged approximately 3.9% (±5.12% SD) of the total study cohort (Figure 8B). Other than the low sample size, the high degree of variation arose due to 5/10 studies reporting no mortality. Overall, the reported mortality matches the known peri-operative mortality rates for domestic rabbits (1.39–4.8%) [59]. Of the four studies that reported mortality events, two were due to complications from anaesthesia and the remaining two did not state a definitive cause.

The larger issue in this case is the lack of unambiguous mortality reporting. While GFSs are extremely safe in humans, and good faith can be assumed in this lack of reporting, it is nonetheless important to return clear survival rates. Unexpectedly large attrition rates in animal studies are an immediate red flag requiring further scrutiny. Aside from animal welfare, a study that reports an emerging technology as safe but only for surviving animals is lacking full scientific rigour. This further feeds into the long running issues regarding translation and reproducibility in pre-clinical studies [60].

More encouragingly, the tracking of general adverse events was significantly more robust, with 70% of the examined studies explicitly detailing these outcomes (Figure 8C). When breaking down the specific nature of these reports (Figure 8D), over half (52.85%) explicitly recorded an absence of any adverse events. Among the documented post-operative complications, hyphema (24%) and general ocular inflammation or irritation (21%) were the most prevalent. A spectrum of less frequent complications was also noted, most prominently corneal edoema (approx. 9%), followed by hypotony and anterior chamber flare (approx. 6% each). Rarer events included infection, bleb leakage, retinal detachment, and tube erosion, which were reported at trace frequencies of roughly 2% or less.

A post-operative complication rate of 47% was in line with rates reported in the Tube Versus Trabeculectomy (TVT) human clinical study (34–57%) [61]. However, results in this clinical trial described outcomes at 12 months, and no timeframe was given for the manifestation of complications beyond that. Additionally, the 24% hyphema rates reported in rabbit GFS studies are considerably higher than the 2–8% reported in the TVT study. This likely reflects the more prominent and exposed ciliary vessel network in rabbit eyes [51] as well as the experimental nature of some interventions.

## 4. Conclusions

The use of animal models in scientific research is justifiably the subject of debate and requires constant scrutiny to ensure ethical and scientific best practice. Currently non-animal alternatives such as organoids and “on-chip” tissues are at advanced stages of development and yielding promising findings [62,63]. However, the field has yet to reach maturity to a degree whereby pre-clinical animal studies can be completely wound down.

For glaucoma filtration surgeries especially, there is little available that can comprehensively replicate the complexity of multiple hydrodynamic systems and a complex inflammatory response following any surgery. As such, it is imperative that pre-clinical rabbit GFS models be designed with the maximum possible clarity and be readily comparable to other studies in the field. This review was undertaken to examine the design, execution and reporting of rabbit GFS studies over a 15-year period across 100 peer-reviewed research articles.

This review found that there were clear commonalities in researcher preference in terms of rabbit breed, surgical technique, post-operative care and post-operative analysis techniques. However, there were some points of note. For example, the tendency for researchers to use very young animals risks the possibility that studies may not be as reflective of human anatomy and physiology as they could be.

As previously stated, the impact of such decisions is unclear. On the face of things, the studies reviewed all succeeded in answering the research question at the heart of the study. However, negative results mostly do not get published. Unlike clinical trials, there is no obligation to report the undertaking of a pre-clinical study. As such, the relative percentage of GFS studies progressing to peer-review is unknown. Additionally, there were frequent incidences of under- or partial reporting of key study data in ethics, animal characteristics, surgery, post-operative care and morbidity and mortality. Discrepancies or omissions typically ranged from 15 to 30% depending on the metric. Reporting practices can be improved with sufficient buy-in from researchers and journal reviewers.

Overall, there is great potential for consensus on GFS in rabbit pre-clinical models without overly limiting any researcher’s ability to design an experiment to meet their needs. It is hoped that this review can provide an idea of some preferences and points to note in rabbit GFS study design and therefore assist in avoiding confounding variables and further increasing cross-comparability between past and future studies.

## Figures and Tables

**Table 1:** Inclusion and exclusion criteria for article selection process.

Inclusion Criteria	Exclusion Criteria
Pre-Clinical Rabbit Model	Literature Outside of Years 2011–2025
English Language	Review Articles/Conference Proceedings or Abstracts
Peer-Reviewed Full Research Article	Canaloplasty (MIGS)
Published 2011–2025 Inclusive	Trabecular By-Pass Shunts (MIGS)
Trabeculectomy Performed	Studies Involving Post-Mortem Rabbits Only
MIBS/GDD Surgery performed	Non-English Language
Glaucoma Filtration Surgery Performed	Non-Rabbit Pre-Clinical Model
No Affiliation to Authors	Human Clinical Model
	No Drainage Surgery Performed (i.e., Conjunctival Flap Formation Only)

## Data Availability

A full list of the reviewed papers is available as Table S1.

## Supplementary Materials

The following supporting information can be downloaded at: https://www.mdpi.com/article/10.3390/vision10030056/s1, Table S1: Full list of the reviewed papers.
