# Supplementary material for: Glaucoma Filtration Surgery in Rabbit Pre-Clinical Models: Design, Drug Dosing and Reporting Practices 2011–2025
Source: Vision (Basel). 2026 Aug 19;10(3):56. doi: 10.3390/vision10030056 (PMC13517750; doi:10.3390/vision10030056)
Supplement: Supplementary file 1 [file vision-10-00056-s001.zip › vision-4403215-supplementary.pdf]

vision

Review

Glaucoma Filtration Surgery in Rabbit Pre-Clinical Models: Design, Drug Dosing and Reporting Practices 2011–2025

Maryam Khan [1,2], Liam Bourke [3], Adrián M. Alambiaga-Caravaca[1], Tauseef Ahmad [1], Mark Lemoine [1], Iluore Asekomhe [1], Golestan Salimbeigi [1], Nina Pohler [1], Colm O'Brien [3,4,5] and Alan J. Hibbitts [1,5,*]

1 Tissue Engineering Research Group, Department Anatomy & Regenerative Medicine, Royal College of Surgeons in Ireland, D02 YN77 Dublin, Ireland
2 Nuffield Laboratory of Ophthalmology, Department of Clinical Neurosciences, University of Oxford, Oxford OX3 9DU, UK
3 Department of Ophthalmology, Mater Misericordiae University Hospital, Eccles Street, D07 R2WY Dublin, Ireland
4 UCD School of Medicine, University College Dublin, Belfield, D04 V1W8 Dublin, Ireland
5 LEP Biomedical Ltd., Nás na Ríogh, W91 HYH9 Cill Dara, Ireland
* Correspondence: alanhibbitts@rcsi.com

Supplementary Material

Supplementary Table S1. Full list of the reviewed papers.

No.	Authors	Year	Title	Link	Paper Discarded	Reason of Discard
1	Swann, Forrest B. MD*; Singh, Sujata MD*; Blake, Diane PhD*,†; John, Vijay PhD‡; Le, Crystal MD*; Fullerton, Mitchell BS*; Margo, Curtis MD§; Zhang, Ze MD‖; Muddasani, Nishika MD*; Wall, Jennifer MD*; Satija, Celine MD*; Greenberg, Erin MD§; Mancera, Norberto MD§; Zurakowski, David PhD¶; Ayyala, Ramesh	2019	Effect of 2 Novel Sustained-release Drug Release Systems on Bleb Fibrosis: An In Vivo Trabeculectomy Study in a Rabbit Model	https://doi.org/10.1097/ijg.0000000000001215	N	
2	Ekinci, Metin MD*; Çagatay, Halil Hüseyin MD†; Ceylan, Erdinç MD‡; Keles, Sadullah MD§; Koban, Yaran MD†; Gokce, Gökçen MD‖; Huseyinoğlu, Urfettin MD¶; Ozcan, Ece MD#; Oba, Mehmet E. MD†	2014	Reduction of Conjunctival Fibrosis After Trabeculectomy Using Topical α-Lipoic Acid in Rabbit Eyes	https://doi.org/10.1097/ijg.0000000000000052	N	
3	Nurettin Sahiner, MS, PhD; Daniel J. Kravitz, MD; Rabah Qadir, MD; Diane A. Blake, PhD; Salima Haque, MD; Vijay T. John, DEngSc; Curtis E. Margo, MD, MPH; Ramesh S. Ayyala, MD, FRCS, FRCOphth	2009	Creation of a Drug-Coated Glaucoma Drainage Device Using Polymer Technology	https://doi.org/10.1001/archophthalmol.2009.19	Y	Date
4	K. Sheng Lim; Sanjeewa S. Wickremasinghe; M. Francesca Cordeiro; Catey Bunce; Peng T. Khaw	2005	Accuracy of Intraocular Pressure Measurements in New Zealand White Rabbits	https://doi.org/10.1167/iovs.04-0610	Y	This paper may be discarded as it is not associated with trab or tube. It studies the intraocular pressure through pressure transducer system in the anterior chamber. Out of date.
5	Ishida K.a · Nakano Y.b · Ojino K.b · Shimazawa M.b · Otsuka T.b · Inagaki S.b · Kawase K.c · Hara H.b · Yamamoto T.c	2020	Evaluation of Bleb Characteristics after Trabeculectomy and Glaucoma Implant Surgery in the Rabbit	https://doi.org/10.1159/000509135	N	
6	Nozomi Igarashi 1, Megumi Honjo 2, Makoto Aihara 1	2021	Effects of mammalian target of rapamycin inhibitors on fibrosis after trabeculectomy	https://doi.org/10.1016/j.exer.2020.108421	N	

7	John Swogger, 1 Ian P. Conner,corresponding author 1 Maranda Rosano, 2 Megan Kemmerer, 2 Carrie Happ-Smith, 1 Alan Wells, 3 , 4 , 5 Joel S. Schuman, 1 , 4 , 5 and Cecelia C. Yatescorresponding author 2 , 3	2020	Injected Versus Sponge-Applied Mitomycin C (MMC) During Modified Trabeculectomy in New Zealand White Rabbit Model	https://doi.org/10.1167/tvst.9.11.23	N	
8	Le-Tien Lin 1 2 3, Jiann-Torng Chen 2 3, Da-Wen Lu 2 3, Ming-Cheng Tai 2 3, Chang-Min Liang 2 3 4, Ching-Long Chen 2 3, Shu-I Pao 3, Chih-Kang Hsu 1 3, Yi-Hao Chen	2020	Antifibrotic role of low-dose mitomycin-c-induced cellular senescence in trabeculectomy models	https://doi.org/10.1371/journal.pone.0234706	N	
9	Maximilian Schultheiss 1, Sven Schnichels 1, Eva-Maria Konrad 1, Karl U Bartz-Schmidt 1, Grit Zahn 2, Patrizia Caldirola 2, Mario G Fsadni 2 3, Ninus Caram-Lelham 2 4, Martin S Spitzer	2017	$\alpha5\beta1$-Integrin inhibitor (CLT-28643) effective in rabbit trabeculectomy model	http://dx.doi.org/10.1111/aos.13215	N	
10	Kuan Jiang 1, Junyi Chen 2, Lingyu Tai 1 3, Chang Liu 1, Xishan Chen 1, Gang Wei 1 4 5, Weiyue Lu 1 4, Weisan Pan 3	2020	Inhibition of post-trabeculectomy fibrosis via topically instilled antisense oligonucleotide complexes co-loaded with fluorouracil	https://doi.org/10.1016/j.apsb.2020.03.002	N	
11	Taiki Kokubun, Kotaro Yamamoto, Kota Sato, Takahiro Akaishi, Atsushi Shimazaki, Masatsugu Nakamura, Yukihiro Shiga, Satoru Tsuda, Kazuko Omodaka, Toru Nakazawa	2019	The effectiveness of colchicine combined with mitomycin C to prolong bleb function in trabeculectomy in rabbits	https://doi.org/10.1371/journal.pone.0213811	N	
12	Hilgert CR, Hilgert AH, Odashiro AN, Odashiro PRP, Paranhos A Jr	2018	Antiscarring effect of intraoperative bevacizumab in experimental glaucoma filtration surgery	https://www.scielo.br/j/abo/a/TvtsgvfFkQcxgNQwgSBBcPr/?lang=en	N	
13	Kunal S. Parikh, Aditya Josyula, Revaz Omiadze, Ju Young Ahn,Youlim Ha, Laura M. Ensign, Justin Hanes, & Ian Pitha	2020	Nano-structured glaucoma drainage implant safely and signifcantly reduces intraocular pressure in rabbits via post-operative outfow modulation	https://doi.org/10.1038/s41598-020-69687-4	N	
14	Cooper D Rodgers, Zachary L Lukowski, Jeff Min, Gina M Martorana, Mary-Kate Wilson, Jamie L Schaefer, Monica A Levine, Craig A Meyers, C Richard Blake, Gregory S Schultz, Mark B Sherwood	2019	Modulating Ocular Scarring in Glaucoma Filtration Surgery Using the Epigenetic Adjunct Suberoylanilide Hydroxamic Acid	https://dx.doi.org/10.5005%2Fjp-journals-10078-1246	N	
15	Christiana Rebello Hilgert, Alvaro Haverroth Hilgert, Alexandre Nakao Odashiro, Patricia Rusa Pereira Odashiro, Augusto Paranhos Jr.	2018	Antiscarring effect of intraoperative bevacizumab in experimental glaucoma filtration surgery	http://dx.doi.org/10.5935/0004-2749.20180062	Y	duplicate
16	Xue Tai, Ying Shen, Haixia Zhao, Zhaoge Wang, Wenying Guan, Xin Kang, Wenqi Guo	2020	Anti-scarring effect of rapamycin following filtering surgery in rabbit eyes		Y	Paper in chinese. Cannot retrieve more than 50% info.

17	Xin Kang, Ying Shen, Haixia Zhao, Zhaoge Wang, Wenying Guan, Ruichun Ge, Ruifang Wang, Xue Tai	2018	Anti-scarring effect of rapamycin in rabbits following glaucoma filtering surgery		Y	Paper in chinese. Cannot retrieve more than 50% info.
18	Nathan A Fischer, Malik Y Kahook, Suhail Abdullah, Eric Porteous, David A Ammar, Jennifer L Patnaik, Jeffrey R SooHoo	2020	Effect of Novel Design Modifications on Fibrotic Encapsulation: An In Vivo Glaucoma Drainage Device Study in a Rabbit Model	https://doi.org/10.1007/s40123-020-00242-0	N	
19	Gong R, Zuo C, Wu K, Zhang S, Qin X, Li Y, Gao X, Haung D, Lin M	2021	A Comparison of Subconjunctival Wound Healing between Different Methods of Dissecting Subconjunctival Tissues	https://doi.org/10.1159/000509551	Y	No Drainage surgery performed
20	Feng Zhang, Ke Liu, Mengdan Cao, Jing Qu, Dengming Zhou, Zheng Pan, Xuanchu Duan, Yong Zhou	2019	Rosiglitazone Treatment Prevents Post operative Fibrosis in a Rabbit Model of Glaucoma Filtration Surgery	https://doi.org/10.1167/iovs.18-26526	N	
21	Wen-Sheng Cheng, Ching-Long Chen, Jian-Torng Chen, Le-Tien Lin, Shu-I Pao, Yi-Hao Chen, Da-Wen LU	2020	AR12286 Alleviates TGF-B Related Myofibroblast Transdifferentiation and Redcues Fibrosis after Glaucoma Filtration Surgery	https://doi.org/10.3390/molecules25194422	N	
22	Yuji Yamamoto, Atushi Mukai, Toru Ikushima, Yasuo Urata, Shingeru Kinoshita, Junji Jamuro, Morio Ueno, Chie Sotozono	2020	Pluripotent epigenetic regulator OBP-801 maintains filtering blebs in glaucoma filtration surgey model	https://doi.org/10.1038/s41598-020-77811-7	N	
23	Birthe SchwerkI, Lisa Harder, Claudia Windho, Marion Hewicker-Trautwein, Anna Wagner, Jan-Peter Bach, Lena Carolin Voigt, Ulf Hinze, Boris Chichkov, Heinz Haferkamp, Holger Lubatschowski, Stephan Nikolic, Ingo Nolte	2019	Comparison of two prototypes of a magnetically adjustable glaucoma implant in rabbits	https://doi.org/10.1371/journal.pone.0215316	N	
24	Kyoung In Jung, Jung Eun Woo, Chan Kee Park	2019	Effects of aqueous suppressants and prostaglandin analogues on early wound healing after glaucoma implant surgery	https://doi.org/10.1038/s41598-019-41790-1	N	
25	Dai Woo Kim, Young Hoon Hwang, Byung Heon Ahn, Eun-Kyung Lee, Chang-Sik Kim	2017	Safety and Efficacy of a Membrane-Tube-Type Glaucoma Shunt Device: An Animal Trial	https://doi.org/10.1080/02713683.2016.1255337	N	

26	Dao-Yi Yu, William H. Morgan, Xinghuai Sun, Er-Ning Su, Stephen J. Cringle,Paula K. Yu, Philip House, Wenyi Guo, Xiaobo Yu	2009	The critical role of the conjunctiva in glaucoma filtration surgery		Y	Paper desrcibes the progress in retinal and Eye research. No experimental research was done.
27	Kenji Matsushita, Rumi Kawashima, Koji Uesugi, Haruka Okada, Hirokazu Sakaguchi, Andrew J. Quantock & Kohji Nishida	2020	Assessment of a self-assembling peptide gel, SPG-178, in providing a clear operative field for trabeculectomy surgery for glaucoma in an animal model	https://doi.org/10.1038/s41598-020-68171-3	N	
28	Blake K Wiliamson, Nathan M Hawkey, Diane A Blake, Joshua W Frenkel, Kevin P McDaniel, Justin K Davis, Celine Satija, Alex Beazer, Suraj Dhungana, James Carlson, Susan McRitchie, Ramesh S Ayyala	2018	The Effects of Glaucoma Drainage Devices on Oxygen Tension, Glycolytic Metabolies, and Metabolimics Porfile of Aqueous Humor in the Rabbit	https://doi.org/10.1167/tvst.7.1.14	N	
29	Feng Zhang, Ke Liu, Zheng Pan, Mengdan Cao, Dengming Zhou, Hairong Liu, Yuting Huang & Xuanchu Duan	2019	Effects of rosiglitazone/PHBV drug delivery system on postoperative fibrosis in rabbit glaucoma filtration surgery model	https://doi.org/10.1080/10717544.2019.1648590	N	
30	Ruken Cinik, Nursen Yuksel, Dilara Pirhan, Mehmet Sechmuz Aslan, Cansu Subasi, Erdal Karaoz	2016	The Effect of Everolimus on Scar Formation in Glaucoma Filtering Surgery in a Rabbit Model	https://doi.org/10.3109/02713683.2015.1125506	N	
31	J L Olson, S Gorman-Lupa	2017	Design and performance of a large lumen glaucoma drainage device		Y	Little experimental data
32	Leaonard Pinchuk, Isabelle Riss, Juan F Batle, Yasushi P Kato, John B Martin, Esdras Arrieta, Paul Palmberg, Richard K Parrish 2nd, Bruce A Weber, Yongmoon Kwon, Jean-Marie Parel	2017	The development of a micro-shunt made from poly(styrene-blocking-isobutylene-block-styrene) to treat glaucoma		Y	No experimental research as paper summarizes the 10 year+ devlopement of InnFocus Micro Shunt
33	Xueru Li, Yu Leng, Xiangji Li, Yawei Wang, Peng Luo, Chi Zhang, Ziwen Wang, Xiaofeng, Yue, Chongxing Shen, Long Chen, Zujuan Liu, Chunmeng Shi, Lin Xie	2020	The TβR II-targeted aptamer S58 prevents fibrosis after glaucoma filtration surgery	https://doi.org/10.18632/aging.102997	N	

34	Li-Jun Cui, Di-Chen Li, Jian Liu, Lei Zhang, Yao Xing	2017	Intraocular pressure control of a novel glaucoma drainage device- in vitro and in vivo studies	https://doi.org/10.18240/ijo.2017.09.04	N	
35	Tatsuo Nagata, Yukinori Harada, Mikki Arai, Tatsuo Hirose, and Hiroyuki Kondo	2020	Polyethylene Glycol-Based Synthetic Hydrogel Sealant for Filtration Bleb Leaks: An In Vivo and Histologic Study	https://doi.org/10.1167/tvst.9.6.24	N	
36	Zhaoxing Dai, Xiaobo Yu, Jianguo Sun, and Xinghuai Sun	2017	Grooved Glaucoma Drainage Devices That Continuously Deliver Cyclosporine A Decrease Postsurgical Scar Formation in Rabbit Eyes	https://doi.org/10.1167/iovs.16-21065	N	
37	Cooper D. Rodgers, Alissa M. Meyer, Nicole C. Rosenberg, Zachary L. Lukowski, Jamie L. Schaefer, Gina M. Martorana, Monica A. Levine, Craig A. Meyers, Mark B. Sherwood	2018	The impact of conjunctival flap method and drainage cannula diameter on bleb survival in the rabbit model	https://doi.org/10.1371/journal.pone.0196968	N	
38	Sabiha Gungor Kobat, Fatma Ulku Celiker, Adile Ferda Dagli, Kader Kasar	2018	The effect on wound healing of pazopanib and bevacizumab compared with corticosteroid in experimental glaucoma filtration surgery	https://doi.org/10.18240/ijo.2018.12.05	N	
39	John Swogger, Ian P. Conner, Maranda Rosano, Megan Kemmerer, Carrie Happ-Smith, Alan Wells, Joel S. Schuman, and Cecelia C. Yates	2020	Injected Versus Sponge-Applied Mitomycin C (MMC) During Modified Trabeculectomy in New Zealand White Rabbit Model	https://doi.org/10.1167/tvst.9.11.23	Y	repeat entry
40	Jeffrey R. SooHoo, Leonard K. Seibold, Ashley E. Laing, Malik Y. Kahook	2012	Bleb morphology and histology in a rabbit model of glaucoma filtration surgery using Ozurdex or mitomycin-C	http://www.ncbi.nlm.nih.gov/pmc/articles/pmc3324358/	N	
41	Jian Ma, Xiaoning Li, Weizhong Zhang, Li Huang, Min Chen, Lei Xi, Zheng Zhang, Lihong Duan, Daping Quan and Jian Ge	2014	CSM Enhances the Filtration Bleb Survival in RabbitModel of Experimental Glaucoma Surgery	https://doi.org/10.3109/02713683.2014.894079	N	
42	Xuan Wang, Ya-Zhi Fan, Liang Yao, Jian-Ming Wang	2016	Anti-proliferative effect of olmesartan on Tenon's capsule fibroblasts	https://doi.org/10.18240/ijo.2016.05.05	Y	No Drainage Surgery
43	Hae-Young L. Park, Jie Hyun Kim, and Chan Kee Park	2013	VEGF Induces TGF-β1 Expression and Myofibroblast Transformation after Glaucoma Surgery	https://doi.org/10.1016/j.ajpath.2013.02.009	N	
44	Marina Hovakimyan, Stefan Siewert, Wolfram Schmidt, Katrin Sternberg,Thomas Reske, Oliver Stachs, Rudolf Guthoff, Andreas Wree, Martin Witt, Klaus-Peter Schmitz, and Reto Allemann	2015	Development of an Experimental Drug Eluting Suprachoroidal Microstent as Glaucoma Drainage Device	https://doi.org/10.1167/tvst.4.3.14	N	

45	Sarah Van de Velde, Tine Van Bergen, Evelien Vandewalle, Nele Kindt, Karolien Castermans, Lieve Moons, Ingeborg Stalmans	2015	Rho kinase inhibitor AMA0526 improves surgical outcome in a rabbit model of glaucoma filtration surgery	https://doi.org/10.1016/bs.pbr.2015.04.014	N	
46	Jayter Silva Paula, Vanessa Raquel Coimbra Ribeiro, Fernando Chahud, Roberta Cannellini, Tassia Cristina Monteiro, Elionai Cassiana de Lima Gomes, Peter Sol Reinach, Maria de Lourdes Veronese Rodrigues, Armnando Silca-Cunha	2013	Bevacizumab-loaded polyurethane subconjunctival implants effects on experimental glaucoma filtration surgery	https://doi.org/10.1089/jop.2012.0136	N	
47	Eun Jung Lee, Jong Chul Han, Do Young Park, Junhun Cho, Changwon Kee	2021	Effect of connective tissue growth factor gene editing using adeno-associaed virus-mediated CRISPR-Cas9 on rabbit glaucoma filtering surgery outcomes	https://doi.org/10.1038/s41434-020-0166-4	N	
48	Li-Fong Seet, Wing Sum Lee, Roseline Su, Sharon N Finger, Jonathon G Crowston, Tina T Wong	2011	Validation of the glaucome filtration surgical mouse model for antifibrotic drug evaluation		Y	The experimental data focused on mouse models
49	Abeer H A Mohamed-Ahmed, Alastair Lockdown, Hala Fadda, Shivam Madaan, Peng T Khaw, Steve Broncchini, Kresti Karu	2018	LC-MS analysis to determine the biodistribution of a polymer coated ilomastat ocular implant		Y	The experiment focused on the effects of ilomastat post surgery
50	Maximilian Schultheiss, Sven Schnichels, Eva-Maria Konrad, Karl U Bartz-Schmidt, Grit Zahn, Patrizia Caldirola, Mario G Fsadni, Ninus Caram-Lelham, Martin S Spitzer	2017	α5β1-Integrin inhibitor (CLT-28643) effective in rabbit trabeculectomy model	http://dx.doi.org/10.1111/aos.13215	Y	repeat entry
51	Sermal Arslan, Organ Aydenur, Mete Guler, Adile Ferda Dagli	2012	Modulation of postoperative scarring with tacrolimus and octreotide in experimental glaucoma filtration surgery	https://doi.org/10.3109/02713683.2011.635404	N	
52	Michael Wilcox, Omar A Kadri	2017	Force and geometry determine structure and function of glaucoma filtration capsules		Y	No surgical data on rabbits
53	Aristides D. Tagalakis, Shivam Madaan, Scott D. Larsen, Richard R. Neubig, Peng T. Khaw, Ian Rodrigues, Saurabh Goyal, Kin Sheng Lim and Cynthia Yu-Wai-Man	2018	In vitro and in vivo delivery of a sustained release nanocarrier-based formulation of an MRTF/SRF inhibitor in conjunctival fbrosis	https://doi.org/10.1186/s12951-018-0425-3	N	
54	Khaled Nassar, Aysegul Tura, Julia Lüke, Matthias Lüke, Salvatore Grisanti, Swaantje Grisanti	2015	A p38 MAPK inhibitor improves outcome after glaucoma filtration surgery	https://doi.org/10.1097/ijg.0000000000000201	N	

55	Abeer H. A. Mohamed-Ahmed; Alastair Lockwood; He Li; Maryse Bailly; Peng T. Khaw; Steve Brocchini	2017	An Ilomastat-CD Eye Drop Formulation to Treat Ocular Scarring		Y	The research study looks into development of topical matrix metalloproteinase inhibitor and does not have information for more than 50% of data required.
56	Kyoung In Jung, Seong-Beom Lee, Jie Hyun Kim, and Chan Kee Park	2013	Foreign Body Reaction in Glaucoma Drainage Implant Surgery	https://doi.org/10.1167/iovs.12-11310	N	
57	Eleni Nikita, Alexandre Moulin, Ioannis Vergados, Dimitrios Brouzas, Panagiotis G. Theodossiadi	2017	A Pilot Study on Ocular Safety and Efficacy of Infliximab as an Antifibrotic Agent After Experimental Glaucoma Filtration Surgery	https://doi.org/10.1007/s40123-017-0096-4	N	
58	Tine Van Bergen,Derek Marshall, Sara Van de Veire, Evelien Vandewalle, Lieve Moons, Jean Herman,Victoria Smith, and Ingeborg Stalmans	2013	The Role of LOX and LOXL2 in Scar Formation After Glaucoma Surgery	https://doi.org/10.1167/iovs.13-11696	N	
59	Zhihong Wu, Shuning Li, Ningli Wang, Wanshun Liu, Wen Liu	2013	A comparative study of the safety and efficacy effect of 5-fluroracil or mitomycin C mounted Biological delivery membranes in a rabbit model of glaucoma filtration surgery	https://doi.org/10.2147/opth.s34200	N	
60	Jung Lim Kim, Hye Sook Lee, Yoonjin Lee, Mi Seon Kang, Sang Joon Lee, Jae Wook Yang	2017	Effect of Porcine Chondrocyte-Derived Extracellular Membrane(CDECM) on Postoperative Wound Healing in an Experimental Rabbit Model of Glaucoma Filtration Surgery	https://doi.org/10.1080/02713683.2016.1257727	N	
61	Jian-Ming Wang, Na Hui, Ya-Zhi Fan, Lei Xong, Nai-Xue Sun	2011	Filtering bleb area and intraocular pressure following subconjunctival injection of GTGF antibody after glaucoma filtration surgery in rabbits	https://doi.org/10.3980/j.issn.2222-3959.2011.05.04	N	

62	Gina M Marorana, Jamie L Schaefer, Monica A Levine, Zachary L Lukowski, Jeff Min, Craig A Meyers, Greogory S Schultz, Mark B Sherwood	2015	Sequential Therapy with Saratin, Bevacizumab and Ilomastat to Prolong Bleb Function following Glaucoma Filtration Surgery in a Rabbit Model	https://doi.org/10.1371/journal.pone.0138054	N	
63	Grierson I, Saheb H, Kahook MY, Johnstone MA, Ahmed II, Schieber AT, Toris CB	2015	A Novel Schlemm's Canal Scaffold: Histologic Observations	https://doi.org/10.1097/ijg.0000000000000012	N	
64	Nikita E, Moulin A, Vergados I, Brouzas D, Theodossiadis PG.	2017	A Pilot Study on Ocular Safety and Efficacy of Infliximab as an Antifibrotic Agent After Experimental Glaucoma Filtration Surgery	https://doi.org/10.1007/s40123-017-0096-4	Y	duplicate
65	Nguyen DQ, Ross CM, Li YQ, Pandav S, Gardiner B, Smith D, How AC, Crowston JG, Coote MA	2012	A model to measure fluid outflow in rabbit capsules post glaucoma implant surgery	https://doi.org/10.1167/iovs.12-10438	N	
66	Villamarin A, Stergiopulos N, Bigler S, Mermoud A, Moulin A, Roy S	2014	In vivo testing of a novel adjustable glaucoma drainage device	https://doi.org/10.1167/iovs.14-14563	N	
67	Shima I, Katsuda S, Ueda Y, Takahashi N, Sasaki H	2007	Expression of matrix metalloproteinases in wound healing after glaucoma filtration surgery in rabbits	https://doi.org/10.1159/000109987	Y	
68	Sun J , Liu X , Lei Y , Tang M , Dai Z , Yang X , Yu X , Yu L , Sun X , Ding J	2017	Sustained subconjunctival delivery of cyclosporine A using thermogelling polymers for glaucoma filtration surgery	https://doi.org/10.1039/c7tb01556a	N	
69	Owen Fernando, Aristides D. Tagalakis, Sahar Awwad, Steve Brocchini,Peng T. Khaw, Stephen L. Hart, and Cynthia Yu-Wai-Man	2018	Development of Targeted siRNA Nanocomplexes to Prevent Fibrosis in Experimental Glaucoma Filtration Surgery	https://doi.org/10.1016/j.ymthe.2018.09.004	N	
70	Hong Ji, Yingting Zhu, Yingying Zhang, Yu Jia, Yiqing Li, Jian Ge and Yehong Zhuo	2017	The Effect of Dry Eye Disease on Scar Formation in Rabbit Glaucoma Filtration Surgery	https://dx.doi.org/10.3390%2Fijms18061150	N	
71	Zhi-chao Yan, Yu-jing Bai, Zhen Tian, Hai-yan Hu, Xiu-hua You, Jian-xian Lin, Shao-rui Liu, Ye-hong Zhuo, Rong-jiang Luo	2011	Anti-proliferation effects of Sirolimus sustained delivery film in rabbit glaucoma filtration surgery	http://www.ncbi.nlm.nih.gov/pmc/articles/pmc3185021/	N	
72	Ashley E. Laing, Leonard K. Seibold, Jeffrey R. SooHoo, Malik Y. Kahook	2012	Evaluation of bleb characteristics after implantation of the EXPRESS™ glaucoma filtration device	http://www.ncbi.nlm.nih.gov/pmc/articles/pmc3253066/	N	

73	Nurettin Sahiner, Daniel J. Kravitz, Rabah Qadir, Diane A. Blake, Salima Haque, Vijay T. John, Curtis E. Margo, Ramesh S. Ayyala	2009	Creation of a Drug-Coated Glaucoma Drainage Device Using Polymer Technology In Vitro and In Vivo Studies	https://doi.org/10.1001/archophthalmol.2009.19	Y	Duplicate Paper
74	Ignacio Rodr[illegible]guez-Agirretxe, Sandra Clarissa Vega, Ricardo Rezola, Elena Vecino, Javier Mendicute, Tatiana Suarez-Cortes, and Arantxa Acera	2013	The PLGA Implant as an Antimitotic Delivery System After Experimental Trabeculectomy	https://doi.org/10.1167/iovs.13-11777	N	
75	Oatts JT, Zhang Z, Tseng H, Shields MB, Sinard JH, Loewen NA	2013	In vitro and in vivo comparison of two suprachoroidal shunts	https://doi.org/10.1167/iovs.13-11853	N	
76	Ichirou Shima, Shogo Katsuda, Yoshimichi Ueda, Nobuo Takahashi, Hiroshi Sasaki	2007	Expression of Matrix Metalloproteinases in Wound Healing after Glaucoma Filtration Surgery in Rabbits		Y	duplicate paper
77	Villamarin A, Roy S, Bigler S, Stergiopulos N.	2014	A new adjustable glaucoma drainage device		Y	Tissue specimens were of post-morem rabbits
78	Pieter Gouws, Edward B. Moss, Graham E. Trope, and C. Ross Ethier	2007	Continuous Intraocular Pressure (IOP) Measurement During Glaucoma Drainage Device Implantation	https://doi.org/10.1097/ijg.0b013e3180391a5d	Y	Pre-2011. Discarded
79	Amoozgar B, Wei X, Hui Lee J, Bloomer M, Zhao Z, Coh P, He F, Luan L, Xie C, Han Y.	2017	A novel flexible microfluidic meshwork to reduce fibrosis in glaucoma surgery	https://doi.org/10.1371/journal.pone.0172556	N	
80	Paschalis EI, Chodosh J, Sperling RA, Salvador-Culla B, Dohlman C	2013	A novel implantable glaucoma valve using ferrofluid	https://doi.org/10.1371/journal.pone.0067404	N	
81	Schoenberg ED, Blake DA, Swann FB, Parlin AW, Zurakowski D, Margo CE, Ponnusamy T, John VT, Ayyala RS	2015	Effect of Two Novel Sustained-Release Drug Delivery Systems on Bleb Fibrosis: An In Vivo Glaucoma Drainage Device Study in a Rabbit Model	https://dx.doi.org/10.1167%2Ftvst.4.3.4	N	
82	Pandav SS, Ross CM, Thattaruthody F, Nada R, Singh N, Gautam N, Beirne S, Wallace GG, Sherwood MB, Crowston JG, Coote M.	2016	Porosity of Bleb Capsule declines rapidly with Fluid Challenge	https://doi.org/10.5005/jp-journals-10008-1208	N	
83	Williamson BK, Hawkey NM, Blake DA, Frenkel JW, McDaniel KP, Davis JK, Satija C, Beazer A, Dhungana S, Carlson J, McRitchie S, Ayyala RS	2018	The Effects of Glaucoma Drainage Devices on Oxygen Tension, Glycolytic Metabolites, and Metabolomics Profile of Aqueous Humor in the Rabbit	https://doi.org/10.1167/tvst.7.1.14	Y	duplicate
84	Min J, Lukowski ZL, Levine MA, Meyers CA, Beattie AR, Schultz GS, Samuelson DA, Sherwood MB	2012	Prevention of ocular scarring post glaucoma filtration surgery using the inflammatory cell and platelet binding modulator saratin in a rabbit model	https://doi.org/10.1371/journal.pone.0035627	N	

85	Xi L, Wang T, Zhao F, Zheng Q, Li X, Luo J, Liu J, Quan D, Ge J	2014	Evaluation of an injectable thermosensitive hydrogel as drug delivery implant for ocular glaucoma surgery	https://doi.org/10.1371/journal.pone.0100632	N	
86	Shobayashi K, Inoue T, Kawai M, Iwao K, Ohira S, Kojima S, Kuroda U, Nakashima K, Tanihara H	2015	Postoperative Changes in Aqueous Monocyte Chemotactic Protein-1 Levels and Bleb Morphology after trabeculectomy vs. Ex-PRESS Shunt Surgery	https://doi.org/10.1371/journal.pone.0139751	N	
87	Lukowski ZL, Min J, Beattie AR, Meyers CA, Levine MA, Stoller G, Schultz GS, Samuelson DA, Sherwood MB	2013	Prevention of ocular scarring after glaucoma filtering surgery using the monoclonal antibody LT1009 (Sonepcizumab) in a rabbit model	https://doi.org/10.1097/ijg.0b013e31822e8c83	N	
88	Zhang C, Wu X, Liu W, Wang J, Guo B, Sun Y, Zhang Y, Zhang H, Wang Y, Zhao J	2013	Impact of application of bio-amniotic membrane immersed in 5-fluorouracil solution in trabeculectomy on rabbit retina	https://doi.org/10.4103/0301-4738.121151	N	
89	Ana C. Acosta, Edgar M. Espana, Hideo Yamamoto, Stewart Davis, Leonard Pinchuk, Bruce A. Weber, Marcia Orozco, Sander Dubovy, Francisco Fantes, Jean-Marie Parel	2006	A newly designed glaucoma drainage implant made of poly(styrene-b-isobutylene-b-styrene): biocompatibility and function in normal rabbit eyes	https://doi.org/10.1001/archopht.124.12.1742; PMID 17159034	Y	date
90	KotaroYamamoto, Taiki Kokubun, Kota Sato, TakahiroAkaishi, Atsushi Shimazaki, Masatsugu Nakamura, Yukihiro Shiga, SatoruTsuda, KazukoOmodaka, Hideyuki Saya & Toru Nakazawa	2019	The DNA topoisomerase II inhibitor amsacrine as a novel candidate adjuvant in a model of glaucoma fltration surgery	https://doi.org/10.1038/s41598-019-55365-7	N	
91	Tomokazu Fujimoto, Kei-Ichi Nakashima, Fumika Watanabe-Kitamura, Takahiro Watanabe, Kenichi Nakamura, Kentaro Maki, Atsushi Shimazaki, Masatomo Kato, Hidenobu Tanihara, and Toshihiro Inoue	2021	Intraocular Pressure-Lowering Effects of Trabeculectomy Versus MicroShunt Insertion in Rabbit Eyes	https://doi.org/10.1167/tvst.10.9.9	N	
92	Virna D. Oktariana Asrory, Ratna Sitompul, Widya Artini, Sri Estuningsih, Deni Noviana, and William H. Morgan	2020	The Inflammatory and Foreign Body Reaction of Polymethyl Methacrylate Glaucoma Drainage Device in the Rabbit Eye	https://doi.org/10.1167/tvst.9.3.20	N	
93	John Swogger, Ian P. Conner, Carrie Happ-Smith, Megan C. Kemmerer, Dana R. Julian, Rachel Davis, Alan Wells, Joel S. Schuman, Cecelia C. Yates+A95:N95	2020	Novel combination therapy reduces subconjunctival fibrosis after glaucoma filtration surgery in the rabbit model	https://doi.org/10.1111/ceo.13884	N	

94	Azadeh Samaeili, Saeed Rahmani, Kiana Hassanpour, Aidin Meshksar, Iman Ansari, Sasha Afsar-Aski, Bahram Einollahi, Mohammad Pakravan+A96:O96	2021	A new glaucoma drainage implant with the use of Polytetrafluoroethylene (PTFE). A pilot study	https://doi.org/10.22336/rjo.2021.30	N	
95	Wang Z, Chen G, Li H, Liu J, Yang Y, Zhao C, Li Y, Shi J, Chen H, Chen G.	2024	Zotarolimus alleviates post-trabeculectomy fibrosis via dual functions of anti-inflammation and regulating AMPK/mTOR axis	10.1016/j.intimp.2024.113176	N	
96	Hsu CC, Lin FP, Tseng HC, Ho PK, Chen YH, Chen YG, Lu DW, Chen YH, Chou JL, Chen HC, Huang YC.	2024	Activation of the ROCK/MYLK Pathway Affects Complex Molecular and Morphological Changes of the Trabecular Meshwork Associated With Ocular Hypertension	10.1167/iovs.65.10.17	Y	no surgery (no trab or tub) is performed
97	Cai M, Luo W, Feng K, Chen Y, Yi L, Zhu X, He J, Liu H, Hutnik C, Wang Y, Li X, Xie L.	2024	Intraocular pressure control efficacy and safety of HA-Mg glaucoma drainage plate implantation in the anterior chamber of rabbit eyes	10.1007/s10856-024-06806-x	N	
98	Wu J, Zhou L, Liu Y, Zhang X, Yang Y, Zhu X, Bu Q, Shan X, Yin J, Weinreb RN, Zhou Q, Pan X, Huang AS.	2024	VEGF-C and 5-Fluorouracil Improve Bleb Survival in a Rabbit Glaucoma Surgery Trabeculectomy Model	10.1167/iovs.65.5.32	N	
99	Hu R, Wang XJ, Chen S, Huang Y, Yu J.	2023	Qingguang'an-induced autophagy in TFs inhibits scar formation: A follow-up in vivo mechanistic investigation	10.1016/j.jtcme.2023.10.002	N	
100	Zhou D, Zhu W, Liu H, Zhang F, Zhou X, Zhang X, Zhao Y, Huang Y, Duan X.	2023	A novel adjustable PHBV basement film for enhancing the efficacy of glaucoma surgery by inhibiting scar formation	10.1016/j.mtbio.2023.100922	N	
101	Ge LY, Wu TH, Liu YQ, Jiang C, Yin X.	2023	Management of experimental trabeculectomy filtering blebs via crosslinking of the scleral flap inhibited vascularization	10.1007/s00417-023-06306-8	N	
102	Jiang C, Yin X, Chang J, Liu G, Lu P.	2023	Topical Administration of Bevacizumab to Facilitate the Functional Filtering Bleb After Trabeculectomy in the Rabbit	10.1089/jop.2023.0046	N	

103	Gowtham L, Halder N, Angmo D, Singh SB, Jayasundar R, Dada T, Velpandian T.	2023	Untargeted metabolomics in the aqueous humor reveals the involvement of TAAR pathway in glaucoma	10.1016/j.exer.2023.109592	Y	no surgery (no trab or tub) is performed
104	Arimura S, Iwasaki K, Neo T, Orii Y, Matsumura T, Takamura Y, Oki M, Inatani M.	2023	Chronic Antioxidant Capacity Loss in Anterior Chamber Environment After Iridectomy	10.1167/tvst.12.5.4	N	
105	Yaakub Azhany, Wan Faiziah Wan Abdul Rahman, Hasnan Jaafar, Jen Hou Low, Wan Nazirah Wan Yusuf, Ahmad-Tajudin Liza-Sharmini, Jemaima Che Hamzah	2023	Clinical and Histopathological Effects of Intracameral Ranibizumab in Experimental Trabeculectomy	10.3390/ijms24087372	N	
106	Refref Yüksel, Nurşen Yüksel, Yusufhan Yazır, Ahmet Öztürk, Selenay Furat Rençber, Kübra Demirci Küçük	2023	The anti-scar effect of tyrosine-kinase inhibitor nintedanib in experimental glaucoma filtration surgery in rabbits	10.1016/j.exer.2023.109431	N	
107	Kiana Hassanpour, Mozhgan Rezaei Kanavi, Narsis Daftarian, Azadeh Samaeili, Fatemeh Suri, Mohammad Pakravan, Azadeh Doozandeh, Sasha Afsar Aski, Maryam Fakhri, Afrooz Moghaddasi, Hamid Ahmadieh, Hamed Esfandiari	2022	The Inhibitory Effect of Connective Tissue Growth Factor Antibody on Postoperative Fibrosis in a Rabbit Model of Trabeculectomy	10.18502/jovr.v17i4.12300	N	
108	Ralph J. S. van Mechelen, Jarno E. J. Wolters, Marjolein Herfs, Christian J. F. Bertens, Marion Gijbels, Leonard Pinchuk, Theo G. M. F. Gorgels, Henny J. M. Beckers	2022	Wound Healing Response After Bleb-Forming Glaucoma Surgery With a SIBS Microshunt in Rabbits	10.1167/tvst.11.8.29	N	
109	Houfa Yin, Xinyi Chen, Xiaogang Hong, Jian Ma, Fang Wu, Ting Wan, Yiwen Sang, Qiuli Fu, Zhenwei Qin, Danni Lyu, Wei Wu, Jinfu Yin, Yabo Yang	2021	Effect of SMILE-derived decellularized lenticules as an adhesion barrier in a rabbit model of glaucoma filtration surgery	10.1186/s12886-021-02090-3	N	
110	Chunlin Lan, Junkai Tan, Li Tang , Guo Liu, Longxiang Huang, Xiaoling Luo, Liang Zhou, Yihua Zhu, Xuyang Liu, Ning Fan	2021	Forkhead domain inhibitory-6 attenuates subconjunctival fibrosis in rabbit model with trabeculectomy	10.1016/j.exer.2021.108725	N	
111	Fumika Watanabe-Kitamura, Akiko Ogawa, Tomokazu Fujimoto, Satoshi Iraha,Miyuki Inoue-Mochita, Takahiro Watanabe, Eri Takahashi, Hidenobu Tanihara,Toshihiro	2021	Potential roles of the IL-6 family in conjunctival fibrosis	10.1016/j.exer.2021.108708	N	
112	Zhu Li Yap, Li-Fong Seet, Stephanie WL Chu, Li Zhen Toh, Farah Ilyana Ibrahim, Tina T Wong	2021	Effect of valproic acid on functional bleb morphology in a rabbit model of minimally invasive surgery	10.1136/bjophthalmol-2020-318691	N	
113	Yazgan, Serpil MD; Tekin, Ishak Ozel MD; Akpolat, Nusret MD; Koc, Ozhan MD	2021	Novel Bacterial Cellulose Membrane to Reduce Fibrosis Following Trabeculectomy	10.1097/IJG.0000000000001907	Y	unable to obtain article

114	Vanessa Andrés-Guerrero,Irene Camacho-Bosca,Liseth Salazar-Quiñones,Nestor Ventura-Abreu,Mercedes Molero-Senosiain,Samuel Hernández-Ruiz,Guillermo Bernal-Sancho,Rocío Herrero-Vanrell and Julián García-Feijóo	2021	The Effect of a Triple Combination of Bevacizumab, Sodium Hyaluronate and a Collagen Matrix Implant in a Trabeculectomy Animal Model	10.3390/pharmaceutics13060896	N	
115	Noa Kapelushnik, Ari Leshno, Reut Singer, Ruth Huna-Baron, Yaniv Barkana & Alon Skaat	2021	Er:YAG laser-assisted filtration surgery: initial results in rabbits	10.1186/s12886-021-01986-4	Y	no trab no tube, LASER
116	K. Kasar, T. Demir , M.M. Akin, S. Gungor Kobat	2021	The effect of halofugenone and pirfenidone on wound healing in experimental glaucoma filtration surgeryL'effet de l'halofugenone et de la perfenedone sur la guérison des plaies en chirurgie de filtration expérimentale du glaucome	10.1016/j.jfo.2020.04.067	N	
117	Kuan Jianga, Junyi Chenb, Lingyu Taia, Chang Liua,Xishan Chena,Gang Weia, Weiyue Lua, Weisan Panc	2020	Inhibition of post-trabeculectomy fibrosis via topically instilled antisense oligonucleotide complexes co-loaded with fluorouracil	10.1016/j.apsb.2020.03.002	Y	repeat
118	Xia Li, Yun Xiao, Xiao-Wei Gao, Yi Liu, Dai-Kun Lei	2020	Experimental study of trabecular tissue repair for corneal defect in rabbits	https://pubmed.ncbi.nlm.nih.gov/32953571/	Y	is trab trasplantation, no trab or tube
119	Kunal S. Parikh, Aditya Josyula, Revaz Omiadze, Ju Young Ahn, Youlim Ha, Laura M. Ensign, Justin Hanes & Ian Pitha	2020	Nano-structured glaucoma drainage implant safely and significantly reduces intraocular pressure in rabbits via post-operative outflow modulation	10.1038/s41598-020-69687-4	Y	duplicate
120	Kenji Matsushita, Rumi Kawashima, Koji Uesugi, Haruka Okada, Hirokazu Sakaguchi, Andrew J. Quantock & Kohji Nishida	2020	Assessment of a self-assembling peptide gel, SPG-178, in providing a clear operative field for trabeculectomy surgery for glaucoma in an animal model	10.1038/s41598-020-68171-3	Y	duplicate
121	Le-Tien Lin, Jiann-Torng Chen, Da-Wen Lu, Ming-Cheng Tai, Chang-Min Liang, Ching-Long Chen, Shu-I Pao, Chih-Kang Hsu, Yi-Hao Chen	2020	Antifibrotic role of low-dose mitomycin-c-induced cellular senescence in trabeculectomy models	10.1371/journal.pone.0234706	Y	Repeat
122	Kyoko Ishida; Yukimichi Nakano; Kazuki Ojino; Masamitsu Shimazawa; Tomohiro Otsuka; Satoshi Inagaki; Kazuhide Kawase; Hideaki Hara; Tetsuya Yamamoto	2021	Evaluation of Bleb Characteristics after Trabeculectomy and Glaucoma Implant Surgery in the Rabbit	10.1159/000509135	Y	Repeat
123	Simar Rajan Singh, Surinder Singh Pandav , Sushmita Kaushik, Ritambhra Nada, Natasha Gautam , Savleen Kaur, Faisal Thattaruthody	2024	Biodegradable material for glaucoma drainage devices – A pilot study in rabbits	10.4103/IJO.IJO_1092_23	N	

124	Michelle T Sun , Renee M Cotton , Chaow Charoenkijkajorn, Julian Garcia-Sanchez , Roopa Dalal , Xin Xia , Jonathan H Lin , Kuldev Singh, Jeffrey L Goldberg , Wendy W Liu	2025	Evaluation of Verteporfin as a Novel Antifibrotic Agent in a Rabbit Model of Glaucoma Filtration Surgery	10.1016/j.xops.2023.100448	N	
125	Chunfang Gan, Shasha Yao, Rugang Pan	2025	Effect of recombinant plasmid heat shock protein 47 small interfering ribonucleic acid on the scarring of the filtering channel after filtration surgery in rabbit eyes	https://doi.org/10.1007/s10792-025-03511-8	N	
126	Jingyuan Liu, Shutong Wang, Guangshuang Tan, Boding Tong, Ying Wu, Lusi Zhang & Bing Jiang	2025	Chitosan-Artesunate nanoparticles: A dual anti-fibrotic and anti-inflammatory strategy for preventing bleb fibrosis post-glaucoma filtration surgery	https://doi.org/10.1007/s13346-025-01819-7	N	
127	Tao Li 1 2, Juan Tang 2 3, Changfen Li 4, Guogang Liu 5, Ying Li 1 2, Shanlan Guo 4, Qilin Fang 1 2, Jing Li 4, Xing Qi 6, Xingde Liu 1 2, Juan Du 1 2, Dan Zhang 1 2, Silun Xiong 1 2, Jiaqian Li 1 2, Yueyue Tan 1 2, Biao Li 1 2, Chuanqiang Dai 7, Qinqin Zhang 7, Jiaman Li 8, Xiaoli Wu 1 2	2025	Evaluating the efficacy and safety of polyglycolic acid-loading mitomycin nanoparticles in inhibiting the scar proliferation after glaucoma filtering surgery	https://doi.org/10.1080/07853890.2024.2436458	Y	Surgery type not specified
128	Susan S Lee 1, Saumya Nagar 1, Lakshmi Rajagopalan 1, Werhner Orilla 1, Karl G Csaky 2, Alexandra Almazan 1, Liuqing Yang 1, Michael R Robinson 1	2024	Using a Novel, Subconjunctival, Sustained-Release Mitomycin C Formulation in a Rabbit Model of Filtration Surgery with Gel Stent Implantation	https://doi.org/10.1089/jop.2023.0100	N	
129	Stefanie Gniesmer # 1, Svenja Rebecca Sonntag # 2, Anna Gapeeva 3, Ala Cojocaru 3 4, Sören Kaps 3, Rainer Adelung 3, Judith Sewing 2, Aysegül Tura 2, Salvatore Grisanti 2, Swaantje Grisanti 2	2024	In vivo evaluation of a nanotechnology-based microshunt for filtering glaucoma surgery	https://doi.org/10.1038/s41598-024-54960-7	N	
130	Zoe Pasvanis 1 2, Roy C K Kong 1 2 3, Elsa C Chan 1 2 3, Jennifer C Fan Gaskin 1 2 4	2025	Inhibitory Effects of 3',4'-Dihydroxyflavonol in a Rabbit Model of Minimally Invasive Bleb Surgery With PreserFlo MicroShunt	https://doi.org/10.1111/ceo.14578	N	
131	Ruiqi Wang 1, Boyang Chen 1, Haiying Wei 2, Wei Yan 2, Yuping Wu 3, Cao Wang 1, Bosong Zhang 1, Fengzhen Liu 4 5, Hui Tian 1, Xiongbiao Chen 6, Weiming Tian 1	2022	Collecting and deactivating TGF-β1 hydrogel for anti-scarring therapy in post-glaucoma filtration surgery	https://www.sciencedirect.com/science/article/pii/S2590006422000588?via%3Dihub	N	n/a

132	Xia Wu 1, Kaili Wu 2, Shibing Ni 3, Shiyi Song 1, Dadong Jia 1, Junjie Tang 4, Jiabing Ran 5, Liang Liang 1	2025	Inhibiting Postoperative Fibrosis in Glaucoma Filtration Surgery Through Porous PLLA/RGD Membrane Mediated Local/Sustained Delivery of Mitomycin C	https://tvst.arvojournals.org/article.aspx?articleid=2811162	N	n/a
133	Yu-Jue Wang 1 2 3, Le-Meng Feng 1 2 3, Cheng Zhang 1 2 3, Wu-Long Zhang 1 2 3, Wei-Tao Song 1 2 3	2022	Anti-scarring effect of sodium hyaluronate at filtration pathway after filtering surgery in rabbits	https://doi.org/10.18240/ijo.2022.04.04	N	n/a
134	Ralph J S van Mechelen 1 2, Jarno E J Wolters 1 2, Sebastian Fredrich 3, Christian J F Bertens 1 2, Marion J J Gijbels 2 3, Albert P H J Schenning 4, Leonard Pinchuk 5, Theo G M F Gorgels 1, Henny J M Beckers 1	2023	A Degradable Sustained-Release Drug Delivery System for Bleb-Forming Glaucoma Surgery	https://doi.org/10.1002/mabi.202300075	N	n/a
135	Yan Liu 1, Chao Gu 1, Xiabin Li 2, Tingwei Wang 3, Ling Yu 4	2021	Involvement of METTL3/m6Adenosine and TGFβ/Smad3 signaling on Tenon's fibroblasts and in a rabbit model of glaucoma surgery	https://doi.org/10.1007/s10735-021-10028-8	N	n/a
